# Maternal plasma lipids are involved in the pathogenesis of preterm birth

**DOI:** 10.1093/gigascience/giac004

**Published:** 2022-02-15

**Authors:** Yile Chen, Bing He, Yu Liu, Max T Aung, Zaira Rosario-Pabón, Carmen M Vélez-Vega, Akram Alshawabkeh, José F Cordero, John D Meeker, Lana X Garmire

**Affiliations:** Department of Computational Medicine and Bioinformatics, University of Michigan, Ann Arbor, MI 48105, USA; Department of Computational Medicine and Bioinformatics, University of Michigan, Ann Arbor, MI 48105, USA; Department of Computational Medicine and Bioinformatics, University of Michigan, Ann Arbor, MI 48105, USA; Program on Reproductive Health and the Environment, Department of Obstetrics, Gynecology, and Reproductive Sciences, University of California, San Francisco, School of Medicine, San Francisco, CA 94158, USA; University of Puerto Rico Graduate School of Public Health, UPR Medical Sciences Campus, San Juan, Puerto Rico 365067, Spain; University of Puerto Rico Graduate School of Public Health, UPR Medical Sciences Campus, San Juan, Puerto Rico 365067, Spain; College of Engineering, Northeastern University, Boston, MA 02115, USA; Department of Epidemiology and Biostatistics, University of Georgia, Athens, GA 30602, USA; Department of Environmental and Health Sciences, School of Public Health, University of Michigan, Ann Arbor, MI 48109, USA; Department of Computational Medicine and Bioinformatics, University of Michigan, Ann Arbor, MI 48105, USA

**Keywords:** preterm, metabolomics, lipid, metabolic pathway, biomarkers, network, fatty acid

## Abstract

**Background:**

Preterm birth is defined by the onset of labor at a gestational age shorter than 37 weeks, and it can lead to premature birth and impose a threat to newborns’ health. The Puerto Rico PROTECT cohort is a well-characterized prospective birth cohort that was designed to investigate environmental and social contributors to preterm birth in Puerto Rico, where preterm birth rates have been elevated in recent decades. To elucidate possible relationships between metabolites and preterm birth in this cohort, we conducted a nested case-control study to conduct untargeted metabolomic characterization of maternal plasma of 31 women who experienced preterm birth and 69 controls who underwent full-term labor at 24–28 gestational weeks.

**Results:**

A total of 333 metabolites were identified and annotated with liquid chromatography/mass spectrometry. Subsequent weighted gene correlation network analysis shows that the fatty acid and carene-enriched module has a significant positive association (*P* = 8e−04, FDR = 0.006) with preterm birth. After controlling for potential clinical confounders, a total of 38 metabolites demonstrated significant changes uniquely associated with preterm birth, where 17 of them were preterm biomarkers. Among 7 machine-learning classifiers, the application of random forest achieved a highly accurate and specific prediction (AUC = 0.92) for preterm birth in testing data, demonstrating their strong potential as biomarkers for preterm births. The 17 preterm biomarkers are involved in cell signaling, lipid metabolism, and lipid peroxidation functions. Additional modeling using only the 19 spontaneous preterm births (sPTB) and controls identifies 16 sPTB markers, with an AUC of 0.89 in testing data. Half of the sPTB overlap with those markers for preterm births. Further causality analysis infers that suberic acid upregulates several fatty acids to promote preterm birth.

**Conclusions:**

Altogether, this study demonstrates the involvement of lipids, particularly fatty acids, in the pathogenesis of preterm birth.

## Introduction

Preterm birth is defined as deliveries that occur prior to 37 weeks of gestation, and it is one of the leading causes of newborn mortality and morbidity [1]. We previously reported that the rates of preterm birth in Puerto Rico are among the highest observed worldwide, reaching 18% [2]. The Puerto Rico PROTECT cohort, herein referred to as the PROTECT cohort, was established to study the etiology of preterm birth and the risk factors associated with it. Factors such as higher maternal age [3], smoking history [4], and lower socioeconomic status, particularly as indicated by education level and income level [5], have been reported to be associated with adverse labor outcome [2]. In addition, we conducted an environmental exposure study in PROTECT and found that higher phthalate exposure was associated with preterm birth [6]. Endogenous metabolites derived from important biological processes (e.g., lipolysis, glycolysis) may provide critical insight into the etiology of antecedent mechanisms of preterm birth [7]; therefore, we conducted a metabolomics study within the PROTECT cohort to establish a potential link between metabolites and preterm birth.

Metabolomics provides compositional and quantitative information about the state of an organism or cell at the macromolecular level [8]. Blood metabolomics has been used to identify biomarkers and potential molecular mechanisms for various diseases and conditions, such as aging [9], acute-on-chronic liver failure [10], hypertension, and blood pressure progression [11]. Biomarkers of preterm birth have been discovered in the amniotic fluid, maternal urine/maternal blood, and cervicovaginal fluid [7]. Decreased phosphocholine (PC) [12] and increased levels of acylglycerophosphoserines (PS), diacylglycerophosphoethanolamines (PE), phosphatidyinositol (PI), and phosphatidylglycerol (PG) were observed in maternal blood samples from women with preterm birth [13]. In a previous lipidomic analysis in the PROTECT cohort, we have also observed signals between maternal free fatty acids (FFAs) and phospholipids (plasmenyl-phosphatidylethanolamines) and spontaneous preterm birth (sPTB) [14]. We sought to expand on this body of evidence and explore greater coverage of metabolic pathways and conducted this study to explore the potential roles that lipids play in preterm birth.

The samples used in this study were maternal plasma collected in gestational weeks 24–28 from the women, who went on to experience preterm birth (N = 31) or full-term healthy deliveries (N = 69). Untargeted metabolomics liquid chromatography with tandem mass spectrometry (LC-MS/MS) assays were performed on these samples, followed by bioinformatics analysis. Our goals were the following: to (i) identify metabolites and metabolomic pathways that are associated with preterm birth; (ii) elucidate metabolomic processes that may have a causal relationship with preterm birth; and (iii) seek early gestational metabolomic biomarkers (weeks 24–28) that are predictive of preterm birth.

## Materials and Methods

### Study population

This study was conducted in an exploratory sample of the PROTECT cohort, which obtained its own institutional review board approval. This is a single-center study conducted in Puerto Rico. At the time of this study, the parent cohort consisted of 812 pregnant women, from which we randomly sampled 31 women who experienced preterm birth and 69 full-term controls for metabolomic analysis. Recruitment of the PROTECT cohort is ongoing and began in 2010. It is funded by the National Institute of Environmental Health Sciences Superfund Research Program. Participants were recruited in the first or second trimester of pregnancy (median 14 weeks gestation). Inclusion during early gestational age ranges allows for greater capacity to evaluate windows of vulnerability across pregnancy. Inclusion criteria for recruitment were as follows: age of 18–40 years, having residence in the Northern Karst aquifer region, disuse of oral contraceptives 3 months before pregnancy, disuse of *in vitro* fertilization, and lack of major health conditions or obstetrical complications in medical records. For preterm births, gestational ages <37 weeks were included. For controls, gestational ages between 39 weeks 0 days and 40 weeks 6 days were included.

### Pregnancy phenotypes

Medical records were used to determine birth outcomes. Gestational age in complete pregnancies was estimated using the American College of Obstetricians and Gynecologists (ACOG) recommendations and previously described in greater detail [6,15,16]. Delivery at <37 weeks gestation was defined to be preterm birth. Among preterm birth cases, we further disaggregated cases as sPTB cases if they had the presentation of premature rupture of membranes, sPTB, or both.

### Sample preparation

Stored plasma samples, which were collected from the women between 24 and 28 weeks gestation and subsequently stored at −80°C, were thawed on ice in preparation for analysis. Deproteinization was then performed by taking 100 µL of plasma combined with 400 µL 1:1:1 ratio of methanol, acetone, and water. Internal standards were also incorporated for metabolite recovery assessment and included 5 µM of L-(D4) thymine, L-(^15^N) anthranilic acid; and 20 µM of L-(^15^N)_2_ tryptophan, gibberellic acid, L-epibrassinolide. Plasma samples were subsequently vortexed and centrifuged for 10 minutes at 15,000*g*. The supernatant of the centrifuged samples was transferred to a clean vial and dried using nitrogen gas. The dried samples were reconstituted to 50 µL.

### Liquid chromatography–mass spectrometry untargeted metabolomics

The untargeted metabolomics analysis of all samples was randomly processed and assigned to LC-MS/MS queue using a computerized algorithm. The reversed-phase chromatographic separation was performed on an Agilent 1290 Infinity II ultra-high performance liquid chromatography instrument (UHPLC) (Agilent Technologies, Inc., Santa Clara, CA, USA) with the Waters Acquity BEH C18 column (Waters Corporation, Milford, MA). The temperature of the column heater was maintained at 55°C. The injection volume was 5 μL for all analyses. The lipid extract was injected onto a 1.7-μm particle diameter, 100 × 2 mm id Waters Acquity BEH C18 column (Waters, Milford, MA) to separate the lipids. We used a linear gradient beginning with 98% Solvent A (water + 0.1% formic acid) and 2% Solvent B (methanol + 0.1% formic acid) to perform chromatographic elution. Solvent B was linearly increased to 98% over the first 22 minutes and was held at this level for 8 minutes. Thereafter, the composition was returned to the beginning and held for 3 minutes. The flow rate used for these experiments was 0.46 mL/min.

Mass spectrometry data acquisition for each sample was performed in both positive and negative ionization modes using an Agilent 6445 Q-TOF (AB Sciex, Concord, ON, Canada). In positive ion mode runs, mobile phase A is 100% water that has 0.1% formic acid while mobile phase B is 100% methanol that has 0.1% formic acid. The formic acid is replaced with 0.1% (m/v) ammonium bicarbonate in negative ion mode runs. The column effluent was directed to the ESI source. For positive ionization mode, the voltage was set to 5,500 V. For negative ionization mode, the voltage was set to 4,500 V. For both modes, the declustering potential (DP) was set to 60 V and the source temperature was set to 450°C. The curtain gas flow was 30 (l/min). The nebulizer was 40 (l/min). The heater gas was 45 (l/min). The Q-TOF resolution according to specifications is >45,000 FWHM at 2,722 *m*/*z*, mass accuracy was <1 ppm with in-line mass calibration, and scan rate was ∼118 scans per minute. Acquisition of MS/MS spectra was performed using the data-dependent acquisition (DDA) function of the Analyst TF software (AB Sciex, Concord, Canada). The software was set to the following parameters: dynamic background subtraction, charge monitoring to exclude multiply charged ions and isotopes, and dynamic exclusion of former target ions for 9 s.

### Metabolite identification

The collected DDA MS/MS spectra data were analyzed using the Masshunter Qualitative Analysis Kit (AB Sciex, Concord, Canada). Using this kit, the “Find by Feature” algorithm is used to detect chromatographic peaks representative of metabolites. Between samples, feature alignment was performed using an in-house–written software package that matches features with identical mass and retention time between samples. Tgaps in chromatographic data, recursive feature identification was also performed by searching the data a second time with the list of aligned features using the “Find by Formula” algorithm in Agilent Masshunter Qualitative Analysis Software. Metabolites were putatively annotated using the mass spectral data annotation tool, Binner [17], to reduce contaminants, artifacts, and degeneracies. An annotated metabolite list was searched against an in-house library of 800 known metabolite standards that had been previously analyzed under identical LC-MS conditions. MS/MS spectra for metabolites not identified by standards were searched in the Metlin (Agilent Metlin B.08.00) or NIST 17. Metabolites not identified by library standards or MS/MS spectra were searched in the Metlin database [47] and Human Metabolome Database (HMDB) [48].

### Metabolomics data pre-processing and quality check

Samples were assayed in a single batch. Pooled quality control (QC) samples were prepared by pooling equal volumes of each sample. The pooled QC samples were injected at the beginning and the end of each analysis and after every 10 sample injections to provide a measurement of the system's stability and performance. The principal component analysis plots of cases, controls, and pool QC samples are shown in Supplementary Fig. S4. A total of 333 metabolite species were detected using the DDA MS/MS spectra data collected either in positive ion mode or negative ion mode. Missing value imputation was performed using the *k*-nearest neighbors method [18]. Log-transformation and quantile normalization [19] was applied to the data, prior to the other downstream analysis. For quality check, partial least squares–discriminant analysis (PLS-DA) was applied on the 100 samples using all identified metabolites.

### Source of variation analysis and data screen

The metabolomics dataset of maternal plasma consists of 333 metabolites, including 167 metabolites in the negative mode and 166 metabolites in the positive mode. To eliminate confounders that are not truly related to preterm birth, we conducted a preliminary screen according to the source of variation (SOV) analysis, which helps to discover the contributions of each clinical/physiological factor to the metabolomics variation. The metabolites with an F statistic of preterm/control >1 were screened before other analyses, meaning that they had a regression sum of squares larger than the error sum of squares. All 333 metabolites passed this screening process.

### Differential metabolomics species identification

To remove potential confounding effects, we fit a linear model for each metabolite over preterm status while adjusting for a priori phenotypic variables via the R limma package [20]. Adjusted phenotypic variables include gestational age in weeks, smoking status, alcohol consumption, baby length, baby (fetal) sex, LGA/SGA (large/small for gestational age), maternal age, income, and pre-pregnancy body mass index (BMI). Large for gestational age (LGA) describes infants that are born with an abnormally high weight, specifically ≥90th percentile, compared to other babies of the same developmental age. Small for gestational age (SGA) describes infants whose weight is <10th percentile for gestational age. Metabolites with *P* < 0.05 were selected as statistically significant in association with preterm birth.

### Weighted gene co-expression network analysis

For the weighted gene co-expression network analysis (WGCNA), all metabolites were analyzed together [21]. The smallest soft threshold with an adjusted *R*^2^ > 0.8 was 4, and hence it was chosen to calculate the adjacency score between any 4 metabolites within a sample set. Following that, the topological overlap value between these 4 metabolites was computed from this adjacency score and the corresponding connectivity value [22]. The topological overlap value is converted to a distance value by subtracting it from 1 and producing a pairwise metabolites distance matrix. This distance matrix was then used to cluster the metabolites using hierarchical clustering with dendrogram, where modules were identified. As a result, we kept the metabolites that had a topological overlap score >0.5 in each module. For the integrated WGCNA analysis using both preterm and healthy samples, we used a soft threshold (power) of 8 as suggested by the WGCNA estimation. We set minModuleSize 10, mergeCutHeight 0.25, deepSplit 2, and verbose 3 for the WGCNA analysis.

### The model of classification

We first further screened the differentiated metabolites with mutual information (MI) >0.5 and then used the Lilikoi package [23] to determine the best machine learning model for classifying preterm and control samples using selected metabolites. Seven algorithms were compared in this step: recursive partitioning and regression trees (RPART), partition around medoids (PAM), gradient boosting (GBM), logistic regression with elastic net regularization (LOG), random forest (RF), support vector machine (SVM), and linear discriminant analysis (LDA). The samples were randomly split into 80/20 ratio for training data vs testing data. The best method was determined on the training set using 10-fold cross-validation, by metrics F statistics and balanced accuracy. We applied the same process above on the subset of 19 sPTB cases and controls.

### The mapping of metabolite-related pathway and phenotype

We used the query lipid as the input to map metabolites to pathways from HMDB, PubChem, and KEGG in Lilikoi [23,24]. These metabolite-pathway interactions were then used for further pathway analysis. Pathway dysregulation scores, a metric representing the degree of dysregulation at the pathway level, were calculated through the Pathifier R package to determine the dysregulation level of the pathway [25].

### Causality analysis

We sorted metabolomics data and clinical features into time series by the gestational ages of patients. Then we performed the Granger causality test to identify potential causality relationships between metabolites and preterm birth using the lmtest R package (version 0.9–37). The threshold of the *P*-value is set to 0.01 for significant causality interaction.

## Results

### Study overview

**Figure 1:**
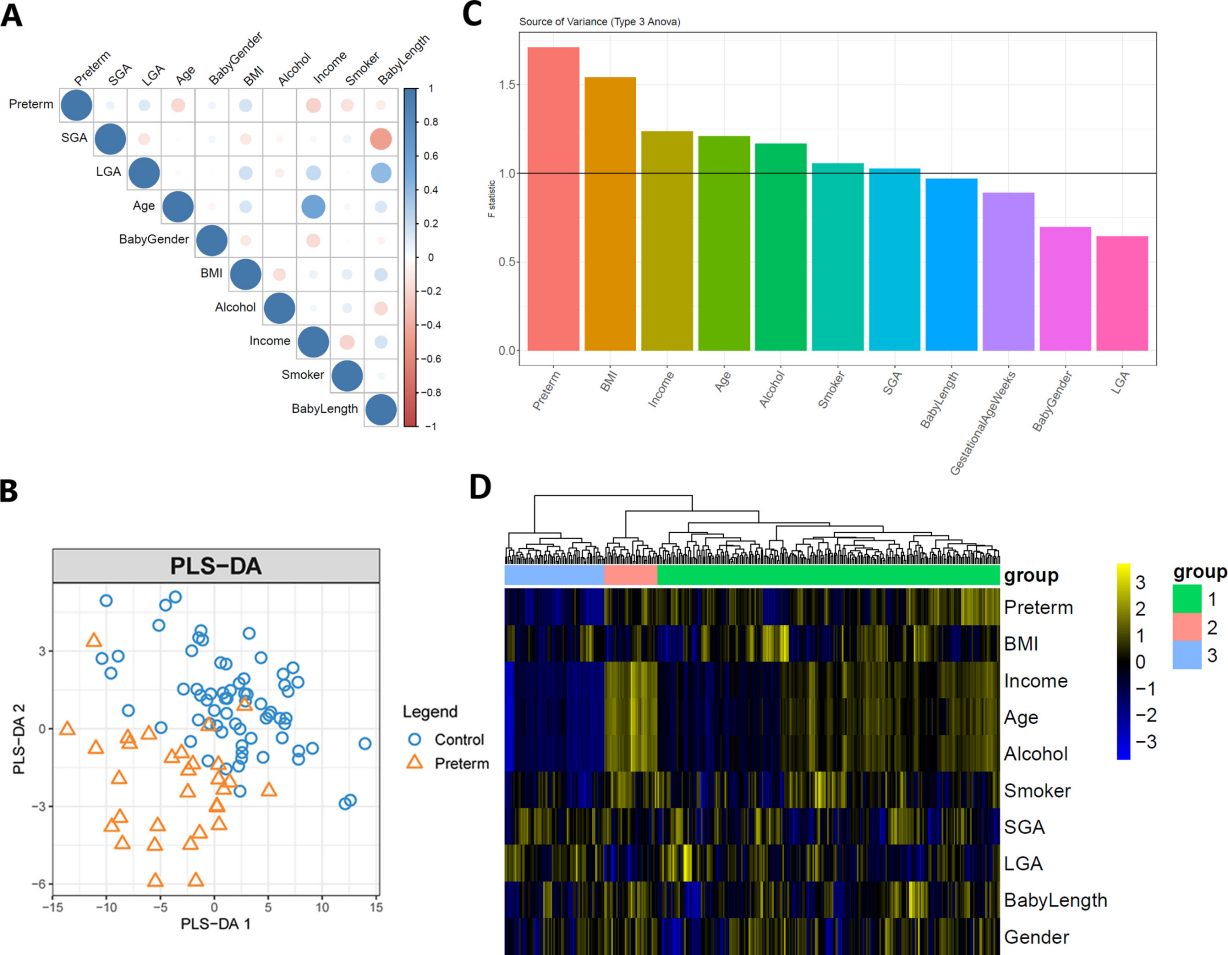
(A) Correlation matrix of the 10 phenotypic variables on the 100 samples (69 controls vs 31 preterm cases). (B) Partial least squares–discriminant analysis (PLS-DA) plot of the 100 samples using 333 metabolites. (C) Source of variation (SOV) analysis using 100 samples; 333 metabolites are used in the ANOVA model. (D) Heat map of correlations between 333 metabolites and 11 confounding factors. The rows represent the clinical factors, and the columns represent metabolites (point-biserial correlation for continuous and binary covariates; Pearson correlation for continuous covariates; Spearman correlation for continuous and ordinal covariates).

The demographic and major clinical characteristics of the participants in the PROTECT cohort study are reported in Table 1. Except for the fact that individuals with preterm deliveries had significantly shorter gestational ages than healthy pregnant women (mean gestational age 39.20 vs 34.69 weeks, *P* = 1.28e−13), other characteristics of cases and controls are comparable across all categories. We also investigated the correlations among phenotypic factors (Fig. 1A). Lower income was positively correlated with preterm birth in weeks (PCC_Income_ = 0.205, *P* < 0.05), confirming the socioeconomic association with preterm birth [26]. Maternal age shows the tendency of negative correlation with preterm birth (PCC_Age_ = −0.181, *P*< 0.1).

**Table 1:** Demographic and clinical characteristics in case and control groups

Characteristic	Controls (n = 69)	Cases (n = 31)	*P*-value^1^
	Mean (SD)		
Maternal age, y	27.07 (5.91)	24.84 (5.10)	0.058
BMI, kg/m^2^	25.55 (5.25)	27.51 (6.92)	0.165
Gestational age, weeks	39.20 (0.98)	34.69 (2.08)	1.28e−13
Annual household income^2^	3.87 (2.12)	2.87 (2.22)	0.039
	No.		
Baby sex			
Female	35	14	0.669
Male	34	17	
Smoker			
Yes	12	2	0.215
No	57	29	
Alcohol use			
None during pregnancy	32	19	0.294
Drank before pregnancy	32	9	
Drank during pregnancy	4	2	
Unknown	1	1	
SGA			
No	58	25	0.567
Yes	10	6	
Unknown	1	0	

1 *t*-test for continuous variables and Fisher exact test for count data.

2 Income categories: 1 = <$4,999; 2 = $5,000–$9,999; 3 = $10,000–$19,999; 4 = $20,000–$29,999; 5 = $30,000–$39,999; 6 = $40,000–$49,999; 7 = $50,000–$74,999; 8 = $75,000–$99,999; 9 = $100,000–$199,999. SGA: small for gestational age.

A total of 333 lipid metabolites were identified by LC/MS in maternal plasma. A PLS-DA plot of the 100 samples using all identified lipid metabolites shows that preterm samples are well separated from healthy controls using the first 2 components (Fig. 1B). To examine the degree of confounding from other variables, an SOV analysis was carried out (Fig. 1C). Preterm birth is ranked first for the F statistics, followed by variables BMI, income, maternal age, alcohol consumption, smoking, and SGA, which all have F statistics >1. To further identify the relationships between phenotypic factors and metabolites, correlations between clinical factors and metabolites were calculated (Fig. 1D) and then subjected to hierarchical clustering (using Euclidean distance as the distance metric). Three clusters of metabolites are identified with sizes of 230, 36, and 67. Cluster 3 is significantly enriched in fatty acids (FAs) (Fisher *P*-value = 5.24e−4, false discovery rate [FDR] = 0.02, odds ratio = 2.12), and FAs are generally lower in preterm samples. They have a striking pattern of negative associations with preterm birth. Moreover, FAs also have overall negative associations with age, income, and alcohol use, suggesting the biological, socioeconomic, and behavioral effects are intertwined at the metabolomic level. The other 2 clusters do not have enrichment in specific metabolite functional groups.

### Correlation network analysis of metabolomics related to preterm birth

**Figure 2:**
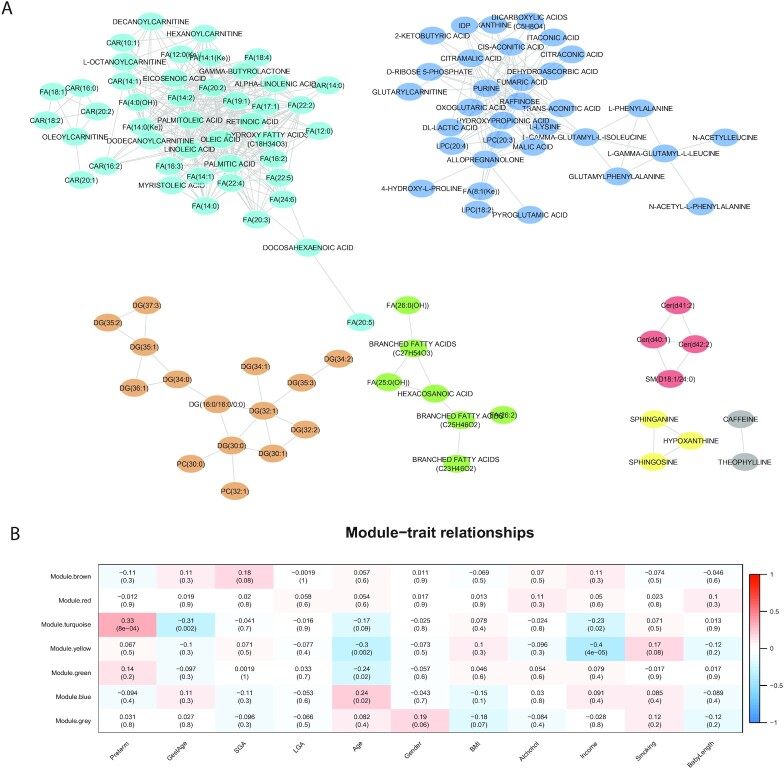
WGCNA network in all samples. (A) WGCNA network modules of metabolomics data from both preterm and control samples. Each node represents a lipid. Node color represents a module. (B) Module-trait associations.

To further elucidate the relationships between metabolomics and preterm birth, we next performed the WGCNA method on the 333 metabolites [21]. WGCNA analysis yields 7 modules (Fig. 2A). Among these modules, only the turquoise-colored module shows a significant positive association (Fisher exact test, *P* = 8e−04, FDR = 0.006) with preterm birth (Fig. 2). This module is enriched with FAs (Fisher exact test, *P* = 3.85e−05, FDR = 4.24e−04) and carene (CAR) (Fisher exact test, *P* = 2.53e−03, FDR = 0.028). This FA/CAR-enriched module also shows a significant negative association (*P* = 0.002, FDR = 0.022) with gestational age (GestAge) (Fig. 2B). These results, together with the previous metabolite-phenotype analysis (Fig. 1C), demonstrate that FAs in the mothers who gave birth prematurely not only have higher levels but also tighter correlations (through regulations). To examine the module difference between cases and controls more closely, we further conducted the WGCNA on the 2 groups separately. Three modules have significantly overlapping metabolites in the case and control groups (Supplementary Figs S1, 2A, and 2B), respectively. Interestingly, the FA-enriched modules in cases (A2) and controls (B2) have the most significant overlap (*P* = 6.76e−18, FDR = 6.084e−17) (Supplementary Figs S1 and 2C). However, we did not find that the density of FA-enriched modules was higher in preterm cases compared to that in controls (Supplementary Figs S1 and 2D).

### Differentiated metabolites and their mapped pathways

**Figure 3:**
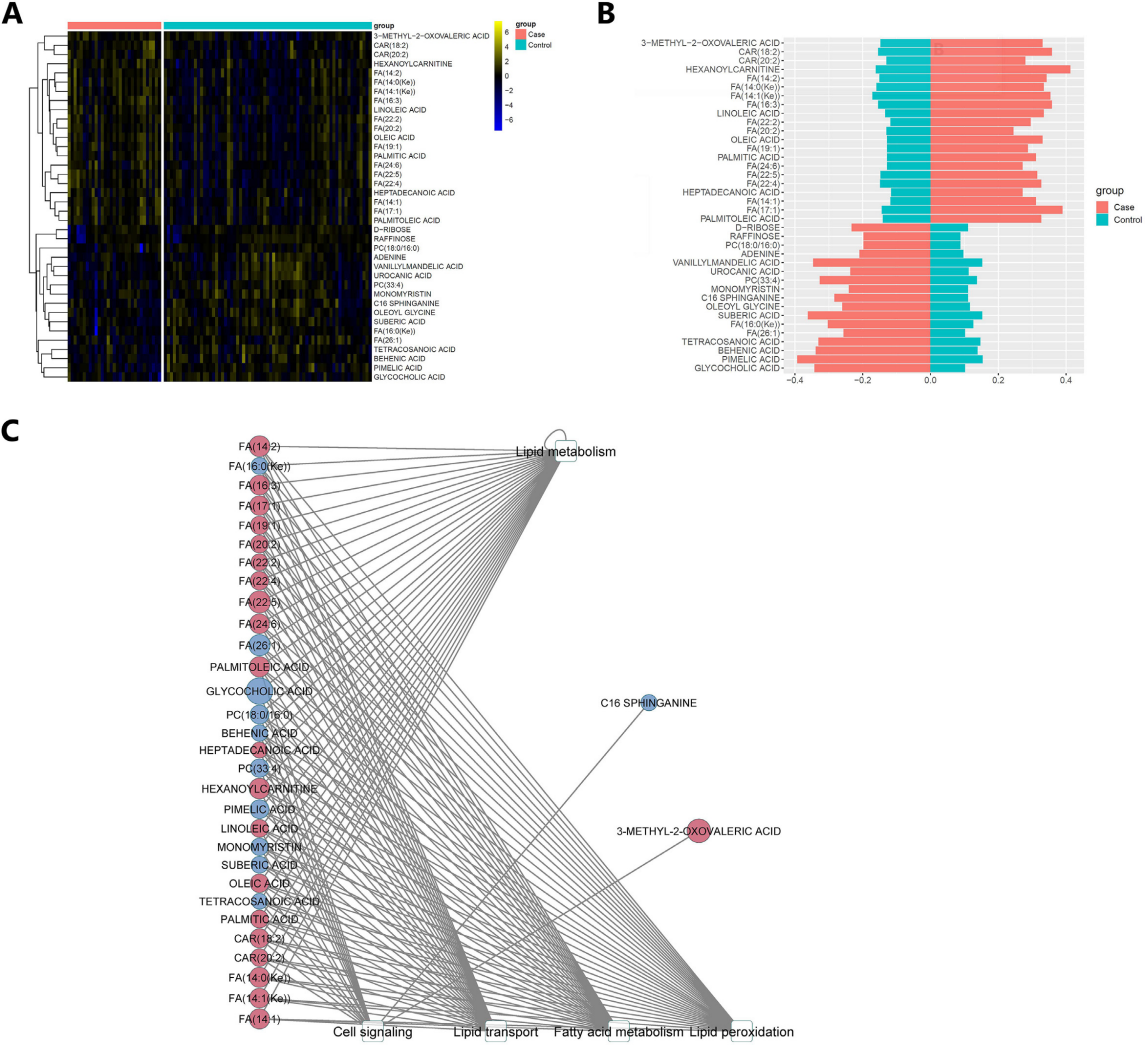
Metabolites show significantly different levels in preterm and control samples. (A) Heat map of the 38 metabolites with a significant difference exclusively between preterm and control samples (*P* < 0.05). (B) Bar plots on the averaged normalized intensities in cases vs controls. (C) Bipartite graph of the significantly differentiated metabolites and the significantly altered metabolic pathways with which they are associated. Five pathways with a significant difference between preterm and control samples (*P* < 0.05) and 33 significantly differentiated metabolites engaged in these pathways are shown. Elliptical nodes: metabolites. Rectangular nodes: pathways from HMDB, PubChem, and KEGG databases. Node color: red, upregulated; blue, down-regulated. Node size: the absolute value of log fold change (logFC).

We next conducted differential metabolite analysis between cases and controls, using the limma package [20] allowing for phenotypic variable adjustment. As a result, 38 metabolites are significantly different (*P* < 0.05) between preterm and control samples exclusively, and are not associated with other confounders (Fig. 3A). The log fold changes (logFC) of the differentiated metabolites ranged from −0.87 to 0.68 (Supplementary Table S1). Among them, 21 metabolites are upregulated and 17 metabolites are downregulated in preterm samples (Fig. 3B). The majority of these metabolites are unsaturated FAs.

To further explore the functions of these metabolites, we mapped the 333 metabolites to pathways and conducted pathway enrichment analysis, using the Lilikoi R package [18,23]. These pathways are from KEGG, HMDB, Metlin, and PubChem databases. A total of 240 of 333 metabolites are successfully mapped by ≥1 database, with assigned memberships to 38 pathways. Among the 38 differential metabolites, 33 of them are involved in 5 pathways that show significant alterations in pathway dysregulation scores, a metric representing the degree of dysregulation at the pathway level [25]. These pathways share a lot of lipids and are interrelated: lipid metabolism, cell signaling, lipid transport, FA metabolism, and lipid peroxidation. The bipartite plot illustrated the relationships between the differentiated metabolites and their corresponding differential pathways (Fig. 3C).

### Metabolomics-based preterm biomarker model

**Figure 4:**
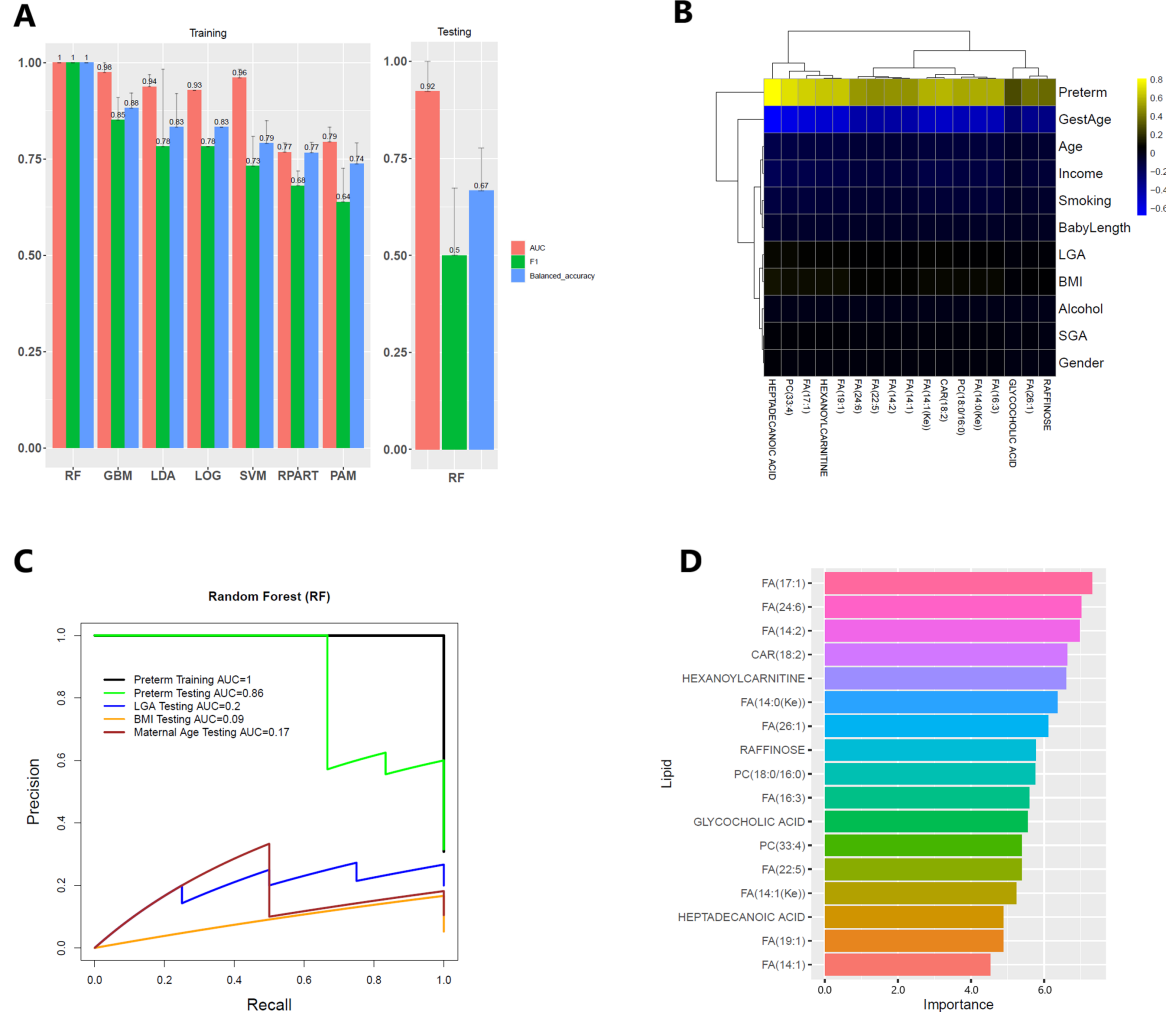
Classification model for preterm birth. (A) Comparison of 7 classification models using 17 metabolites on the hold-out testing. The dataset was randomly split into training data (80%) and testing data (20%) 10 times. The mean value (bars) and standard error (error bars) of the 10 repeats are shown for 3 performance metrics of the area under the receiver operating curve (AUC), F1 statistic, and balanced accuracy. The winning method RF in training data (left) was then applied to the testing data (right). (B) The heat map of correlation coefficients between the 17 metabolites and clinical variables. (C) The precision-recall curves of the RF model from (A) on classifying preterm, LGA (large for gestational age), income, and maternal age (≥35 y or not), respectively, using the same set of testing data as in (A). (D) Normalized variable importance scores for the 17 lipid markers in the RF model. The normalization is done on R by making the sum of importance scores be 100.

Another important application of metabolomics analysis is to screen for diagnostic biomarkers for diseases. For this purpose, we split samples with 80/20 ratio into training and testing data. We further selected 17 metabolites out of the 38 differentiated ones using MI score of 0.5 as the threshold. We compared the performance of 7 machine learning algorithms in the Lilikoi R package, including RPART, PAM, GBM, LOG, RF, SVM, and LDA. We used the area under the ROC curve (AUC), F1 statistic, and balanced accuracy to evaluate the models. Among all classification methods, RF yields the highest balanced accuracy statistic (1.0) in the training dataset (Fig. 4A), so we selected it as the winning model to show the predictive performance on the remaining testing dataset. The overall accuracy for RF on the testing data is 0.92 for the AUC, 0.5 for the F1 statistic, and 0.67 for the balanced accuracy (Fig. 4C). Next, we tested whether the biomarkers are specific to preterm birth rather than other clinical confounders. We used the 17-feature RF classification model built for preterm birth to predict its classification performance over other terms including LGA, BMI, and maternal age, using the same testing dataset. The AUC on LGA, BMI, and maternal age are 0.2, 0.09, and 0.17 respectively, in the precision-recall curves (Fig. 4D). This confirms the specificity of the 17-biomarker model for preterm birth. Several FAs show top importance scores in the model: FA(17:1) (first, importance score = 7.32 of 100); FA(24:6) (second, 7.02); FA14:2 (third, 6.98). Hexanoylcarnitine is also a top important metabolite (fifth, 6.6), involved in FA oxidation. It has been reported to be significantly higher in preterm birth [27].

### Predicted causality interactions among metabolites and preterm birth

**Figure 5:**
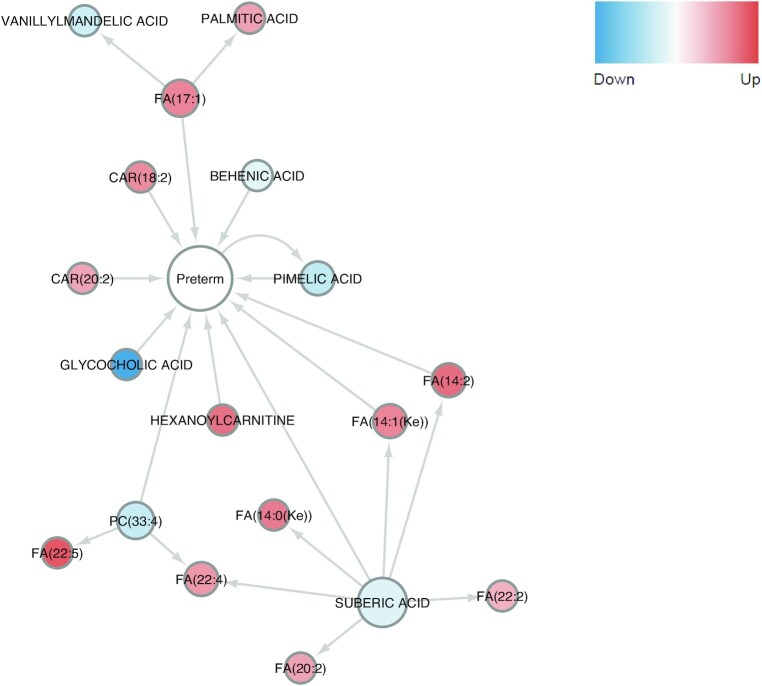
Predicted significant (*P* < 0.01) causality interactions between the 17 metabolites and preterm birth. Arrow indicates the causality interaction. Blue and red nodes are down- and upregulated metabolites, while the center one is preterm.

We used the Granger causality test [28] to infer significant causality interactions (*P* < 0.01) between the 17 metabolites and the binary preterm outcome. As shown in Fig. 5, upregulated hexanoylcarnitine (logFC = 0.472), CAR(18:2) (logFC = 0.375), CAR(20:2) (logFC = 0.280), FA(14:1(Ke)) (logFC = 0.407), FA(14:2) (logFC = 0.492), and FA(17:1) (logFC = 0.402), and downregulated behenic acid (logFC = −0.191), pimelic acid (logFC = −0.357), suberic acid (logFC = −0.224), glycocholic acid (logFC = −0.867), and PC(33:4) (logFC = −0.332) are predicted as direct causal metabolites of preterm birth. The causality test also predicts the causality interaction from FA(17:1) to pimelic acid, which is synthesized from FA [29]. Interestingly, downregulated suberic acid (logFC = −0.224) is predicted to be the direct cause of upregulated FA(22:4) (logFC = 0.332), FA(20:2) (logFC = 0.282), FA(22:2) (logFC = 0.221), FA(14:0(Ke)) (logFC = 0.434), FA(14:1(Ke)) (logFC = 0.407), and FA(14:2) (logFC = 0.492). A previous study shows that suberic acid is present in the urine of patients with FA oxidation disorders, indicating the correlation between suberic acid and the metabolism of FAs [30].

### Prediction model for spontaneous preterm birth

The 31 preterm samples include 19 sPTB cases and 12 samples from other conditions (e.g., preeclampsia). To further investigate the association between metabolites and sPTB, we analyzed the cases with sPTB separately (cases = 19; controls = 75). We conducted differential analysis between sPTB and controls and identified 53 metabolites with *P* < 0.05, 33 of which also appeared in the previous 38 metabolites significantly different in preterms vs controls (Supplementary Fig. S2A). For the 33 metabolites, the differential patterns are consistent in both preterm birth and sPTB, with the fold changes being more extreme in sPTB (Supplementary Fig. S2B).

Using the same procedures as in the previous metabolomics-based preterm biomarker model, we identified 16 of the 55 metabolites as markers for sPTB. Half of these 16 markers, including FA(24:6), FA(16:3), FA(17:1), FA(14:2), FA(19:1), FA(14:0(Ke)), FA(14:1), and heptadecanoic acid, are also among the previously identified 17 metabolite markers for preterm birth. We further investigated whether the identified markers can serve as good predictors of sPTB (Supplementary Fig. S3). The RF method again presents the best performance in the training data and achieves an AUC of 0.89 in the testing data. In the metabolite marker importance ranking, previously fifth-ranked hexanoylcarnitine appears again among the top metabolites (fifth, 7.56). In summary, many preterm markers are also robust sPTB signatures.

## Discussion

Preterm birth is one of the leading causes of newborn mortality and morbidity [1]. To improve our understanding of preterm birth, we conducted a metabolomics analysis of maternal blood in the PROTECT cohort of preterm birth patients and healthy controls.

The importance of FAs in preterm birth is highlighted by bioinformatics analysis in various aspects. First, correlation network analysis of metabolomics reveals deregulated lipid modules that may contribute to preterm birth (Fig. 2). The FA/CAR-enriched module is enriched with several FAs including 2 essential FAs, i.e., alpha-linolenic acid and linoleic acid (omega-6 FA), and a class of saturated FAs (heptadecanoic acid, palmitic acid). Second, FAs show high importance scores in machine learning models for either preterm birth or sPTB. Other studies have also found excessive FFAs detected in the preterm cases of maternal circulation, linking them to inflammation [31], the main cause of preterm birth [27]. In fact, a higher omega-6 to omega-3 FA ratio would increase pro-inflammatory eicosanoid production [32,33], and it was associated with shorter gestation duration for overweight/obese women [34]. Another study on underweight and obese women with sPTB identified a higher concentration of omega-6 and omega-3 FAs in their mid-gestation serum samples [35]. Confirming our discovery, a recent complementary lipidomics study within the PROTECT cohort also observed that mono- and polyunsaturated FFAs (FFA 20:1, FFA 20:1, FFA 18:1) were associated with a higher risk of sPTB [14]. We have also found complementary evidence in the LIFECODES cohort of positive associations between sPTB and eicosanoids, which are secondary metabolites of polyunsaturated FA parent compounds such as arachidonic acid [36]. Besides FAs, 2 phosphatidylcholine (PC(18:0/16:0), PC(33:4)) were also selected by the biomarker model for preterm birth. These 2 metabolites have lower levels in preterm births. PCs are the main structure of cell membranes and play an important role in maintaining membrane stability and reducing inflammation [37]. Consistent with this, 1 recent study also found a class of PC significantly lower in preterm births [38].

Interestingly, the causality analysis shows the causal effect of decreased suberic acid for the excessive FAs. This is consistent with a previous finding that suberic acid is related to FA disorders [30]. Suberic acid, also called octanedioic acid, is a dicarboxylic acid, which can be produced from FAs [39]. The production from FAs to dicarboxylic acids is catalyzed by cytochrome P450 (CYP) 4 F/A (CYP4F/A) enzymes [39,40]. The accumulation of FAs and reduction of suberic acid in preterm maternal blood samples (Fig. 5) suggest that CYP4F/A enzymes, the enzyme catalyzing this conversion, have reduced activities in preterm delivery. Polymorphisms in CYP4F/A genes, which impair enzyme functions, previously showed associations with preterm birth [41]. Thus, we speculate that polymorphisms or other forms of deactivation of CYP4F/A genes may play a role in preterm births.

**Figure 6:**
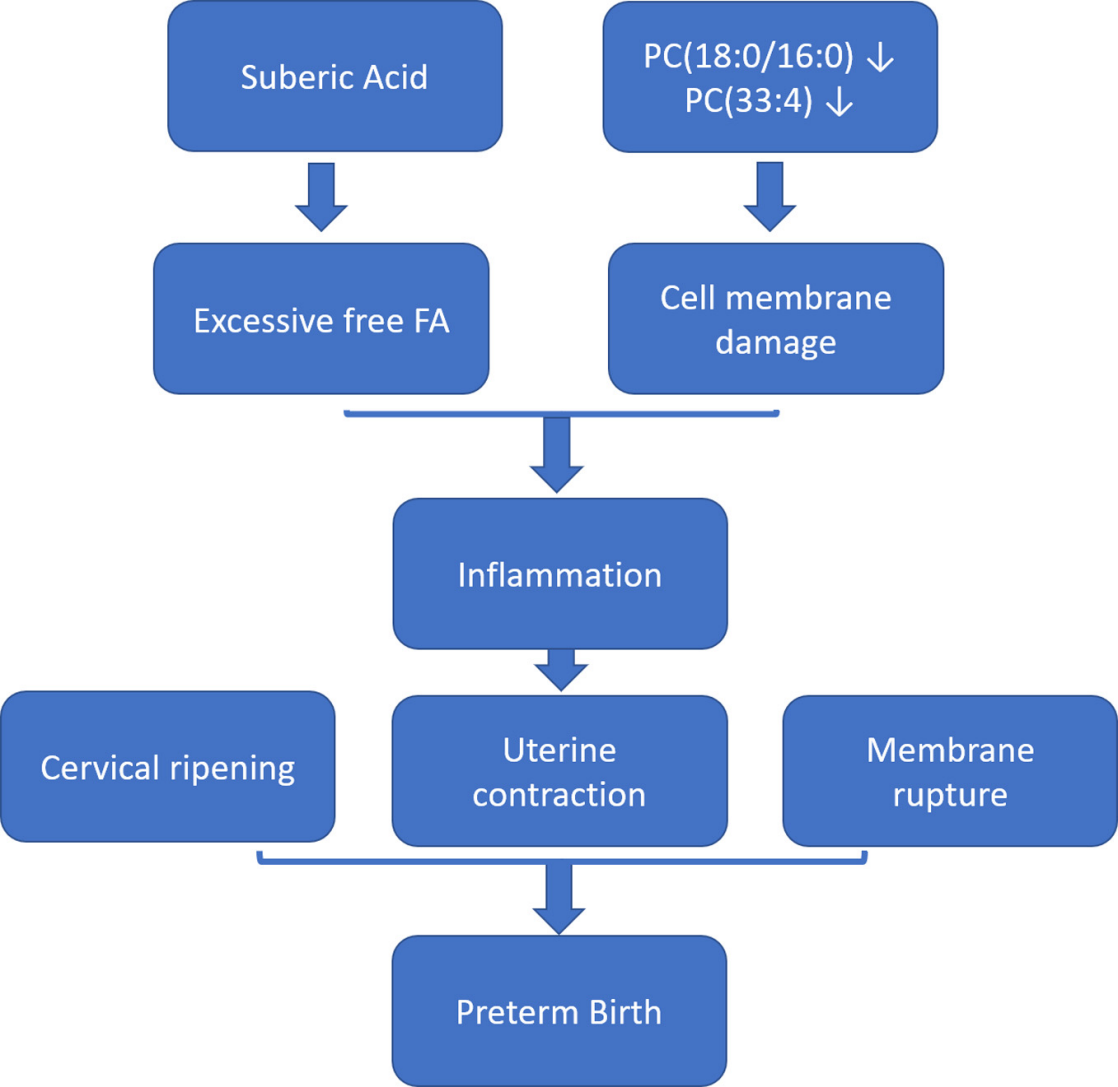
A proposed model of metabolite changes affecting preterm birth.

Changes in these lipids collectively suggest that lipid metabolism may contribute to the pathogenesis of preterm birth (Fig. 6). Indeed, several related pathways including lipid metabolism, FA metabolism, and lipid peroxidation pathways are all enriched in the preterm cases (Fig. 3C). These pathways were discussed frequently in many previous preterm birth analyses [38,42,43]. Unsaturated FAs, shown to be excessive in preterm samples of this dataset, are more likely to undergo lipid peroxidation [44]. Unsaturated FAs and the evident lipid peroxidation process could lead to oxidative stress, which was reportedly related to preterm birth through regulating cervical ripening, uterine contraction, and membrane rupture [42]. In addition, accelerated lipid peroxidation is found in prematurity [45].

A few caveats of this study should also be mentioned. First, participants in this study do not have specific dietary records; thus potential confounding from diet cannot be investigated. The metabolites are measured from maternal blood; therefore any biological mechanisms discussed here are inferred systematically rather than being directly measurable from relevant tissues (e.g., placenta). In addition, despite developing a potential biomarker panel from a classification model, these candidates are suggestive and not quantitatively validated yet. We plan to validate them in other independent cohorts in the future. Nonetheless, this study provides strong evidence of the involvement of a class of saturated and unsaturated FAs and PCs in preterm births, mediated by perturbation in biological functions including cell signaling and lipid peroxidation.

## Availability of Source Code and Requirements

Project name: Maternal lipids in the pathogenesis of preterm birth

Project home page: https://github.com/lanagarmire/pretermBirth_metabolomics

Operating systems: Windows and Linux

Programming language: R

License: MIT

## Data Availability

These data are available at the NIH Common Fund's National Metabolomics Data Repository (NMDR) website, the Metabolomics Workbench, https://www.metabolomicsworkbench.org, where they have been assigned Project ID PR001155. The data can be accessed directly via their Project DOI: http://dx.doi.org/10.21228/M8DH5P. Other data further supporting this work are openly available in the *GigaScience* repository, GigaDB [46].

## Additional Files

**Supplementary Figure S1**. (A, B) WGCNA network in preterm births (A) and healthy controls (B), respectively. Each node represents a metabolite, whose size is proportional to the node connectivity value in a WGCNA network. (C) The overlap between modules of networks in control and preterm samples. (D) Detailed information on overlapping module density was discovered in (C). (E) Bar plot of the connectivity scores of the 17 upregulated metabolites.

**Supplementary Figure S2**. Metabolites show significantly different levels in sPTB and control samples. (A) Heat map of the 55 metabolites with a significant difference exclusively between sPTB and control samples (*P* < 0.05). (B) Bar plots on the averaged normalized intensities in cases vs controls.

**Supplementary Figure S3**. Classification model for sPTB. (A) Comparison of 7 classification models using 16 metabolites on the hold-out testing. The dataset was randomly split into training data (80%) and testing data (20%) 10 times. The average value and standard error of the 10 repeats are shown for 3 performance metrics of the area under the ROC curve (AUC), F1 statistic, and balanced accuracy. The winning method RF in training data (left) was then applied to the testing data (right). (B) The heat map of correlation coefficients between the 16 metabolites and clinical variables. (C) The precision-recall curves of the RF model from (A) on classifying preterm, LGA (large for gestational age), income, and maternal age (≥35 y or not), respectively, using the same set of testing data as in (A). (D) Normalized variable importance scores for the 16 lipid markers in the RF model. The normalization is done by making the sum of importance scores equal 100.

**Supplementary Figure S4**. Principal component analysis plots for the QC of the metabolomics data in positive and negative modes.

**Supplementary Table S1**. Fold change values of the 38 metabolites that are significantly different between preterm and control samples.

giac004_GIGA-D-21-00205_Original_Submission

giac004_GIGA-D-21-00205_Revision_1

giac004_GIGA-D-21-00205_Revision_2

giac004_Response_to_Reviewer_Comments_Revision_1

giac004_Reviewer_1_Report_Original_Submission Renato T Souza -- 7/24/2021 Reviewed

giac004_Reviewer_2_Report_Original_Submission Xiangping Lin -- 8/25/2021 Reviewed

giac004_Reviewer_2_Report_Revision_1 Xiangping Lin -- 11/22/2021 Reviewed

giac004_Supplemental_Figures_and_Table

## Abbreviations

ANOVA: analysis of variance; AUC: area under the receiver operating curve; CAR: carene; CYP4F/A: cytochrome P450 (CYP) 4 F/A; FA: fatty acid; FDR: false discovery rate; FWHM: full width at half-maximum; GBM: gradient boosting; HMDB: Human Metabolome Database; KEGG: Kyoto Encyclopedia of Genes and Genomes; LC-MS/MS: liquid chromatography with tandem mass spectrometry; LDA: linear discriminant analysis; LGA: large for gestational age; LOG: logistic regression with elastic net regularization; MI: mutual information; NIH: National Institutes of Health; PAM: partition around medoids; PC: phosphocholine; PE: diacylglycerophosphoethanolamines; PG: phosphatidylglycerol; PI: phosphatidyinositol; PS: acylglycerophosphoserines; QC: quality control; Q-TOF: quadrupole time-of-flight; RF: random forest; RPART: recursive partitioning and regression trees; SGA: small for gestational age; SOV: source of variation; SVM: support vector machine; WGCNA: weighted gene correlation network analysis.

## Competing Interests

The authors declare that they have no competing interests.

## Funding

This study was supported by the Superfund Research Program of the National Institute of Environmental Health Sciences, National Institutes of Health (grants P42ES017198). Additional support was provided from NIEHS grant numbers P50ES026049, R01ES032203, and P30ES017885 and the Environmental influences on Child Health Outcomes (ECHO) program grant NO. UH3OD023251, and the NIH grant U2C-DK119886. L.X.G. is supported by grants K01ES025434 awarded by NIEHS through funds provided by the trans-NIH Big Data to Knowledge (BD2K) initiative, R01 LM012373, and LM012907 awarded by NLM, and R01 HD084633 (L.X.G.) awarded by NICHD.

## Authors' Contributions

Y.C. and B.H. conducted the bioinformatics analysis and modified code provided by Y.L. M.T.A. provided writing material. J.D.M. designed the study, obtained funding, supervised the metabolomics assays, and critically reviewed early drafts of the paper. L.X.G. supervised the analysis. Y.C., B.H., and L.X.G. wrote the manuscript. All authors have read and revised the manuscript.

## Conflicts of Interst

The authors declare that they have no competing interests.
