# Supplementary material for: Maternal plasma lipids are involved in the pathogenesis of preterm birth
Source: Gigascience. 2022 Feb 15;11:giac004. doi: 10.1093/gigascience/giac004 (PMC8847704; doi:10.1093/gigascience/giac004)
Supplement: giac004_GIGA-D-21-00205_Revision_2 [file giac004_giga-d-21-00205_revision_2.pdf]

GigaScience

Maternal plasma lipids are involved in the pathogenesis of preterm birth

--Manuscript Draft--

**Manuscript Number:**	GIGA-D-21-00205R2
**Full Title:**	Maternal plasma lipids are involved in the pathogenesis of preterm birth
**Article Type:**	Research
**Funding Information:**	national institutes of health (P42ES017198) — Dr John D. Meeker<br>national institutes of health (P50ES026049) — Dr John D. Meeker<br>national institutes of health (R01ES032203) — Dr John D. Meeker<br>national institutes of health (P30ES017885) — Dr John D. Meeker<br>national institutes of health (K01ES025434) — Dr Lana Garmire<br>national institutes of health (R01LM012373) — Dr Lana Garmire<br>national institutes of health (R01LM012907) — Dr Lana Garmire<br>national institutes of health (R01HD084633) — Dr Lana Garmire

Abstract:

Background

Preterm birth is defined by the onset of labor at a gestational age shorter than 37 weeks and it can lead to premature birth and impose a threat to newborns' health. The Puerto Rico PROTECT cohort is a well-characterized prospective birth cohort that was designed to investigate environmental and social contributors to preterm birth in Puerto Rico, where preterm birth rates have been elevated in recent decades. To elucidate possible relationships between metabolites and preterm birth in this cohort, we conducted a nested case-control study to conduct untargeted metabolomic characterization of maternal plasma of 31 preterm birth women and 69 full-term labor controls at 24-28 gestational weeks.

Results

A total of 333 metabolites were identified and annotated with liquid chromatography/mass spectrometry. Subsequent weighted gene correlation network analysis shows the fatty acid and carene enriched module has a significant positive association (p-value=8e-04, FDR=0.006) with preterm birth. After controlling for potential clinical confounders, a total of 38 metabolites demonstrated significant changes uniquely associated with preterm birth, where 17 of them were preterm biomarkers. Among seven machine-learning classifiers, the application of random forest achieved the highly accurate and specific prediction (AUC = 0.92) for preterm birth in testing data, demonstrating their strong potential as biomarkers for preterm births. The 17 preterm biomarkers are involved in cell signaling, lipid metabolism, and lipid peroxidation functions. Additional modeling using only the 19 spontaneous preterm births (sPTB) and controls identifies 16 sPTB markers, with an AUC of 0.89 in testing data. Half of the sPTB overlaps with those markers for preterm births. Further causality analysis infers that suberic acid upregulates several fatty acids to promote preterm birth.

Conclusions

Altogether, this study demonstrates the involvement of lipids, particularly fatty acids, in the pathogenesis of preterm birth.

Corresponding Author: Lana Garmire

UNITED STATES

Powered by Editorial Manager® and ProduXion Manager® from Aries Systems Corporation

Corresponding Author Secondary Information:	
Corresponding Author's Institution:	
Corresponding Author's Secondary Institution:	
First Author:	Yile Chen
First Author Secondary Information:	
Order of Authors:	Yile Chen
	Bing He
	Yu Liu
	Max T. Aung
	Zaira Rosario-Pabón
	Carmen M. Vélez-Vega
	Akram Alshawabkeh
	José F. Cordero
	John D. Meeker
	Lana Garmire
Order of Authors Secondary Information:	
Response to Reviewers:	Dear Editor,<br>Thanks for pointing out the potential misleading description in the method section. "The same gradient and solvent system were used for positive and negative modes of MS analysis. ". The term "same" here means the gradient. Solvent A and B are same as the previous paragraph. It's a bit misleading here so we deleted this sentence, since the following sentence describe the solvent A and B again.<br>Thanks<br>Lana
Additional Information:	
**Question**	**Response**
Are you submitting this manuscript to a special series or article collection?	No
**Experimental design and statistics**<br><br>Full details of the experimental design and statistical methods used should be given in the Methods section, as detailed in our Minimum Standards Reporting Checklist. Information essential to interpreting the data presented should be made available in the figure legends.<br><br>Have you included all the information requested in your manuscript?	Yes
**Resources**	Yes

A description of all resources used, including antibodies, cell lines, animals and software tools, with enough information to allow them to be uniquely identified, should be included in the Methods section. Authors are strongly encouraged to cite Research Resource Identifiers (RRIDs) for antibodies, model organisms and tools, where possible.<br><br>Have you included the information requested as detailed in our Minimum Standards Reporting Checklist?	
**Availability of data and materials**<br><br>All datasets and code on which the conclusions of the paper rely must be either included in your submission or deposited in publicly available repositories (where available and ethically appropriate), referencing such data using a unique identifier in the references and in the “Availability of Data and Materials” section of your manuscript.<br><br>Have you have met the above requirement as detailed in our Minimum Standards Reporting Checklist?	Yes

Powered by Editorial Manager® and ProduXion Manager® from Aries Systems Corporation

Manuscript with DOI

Click here to access/download;Manuscript;Preterm project manuscript_minorRevision with DOI.docx

Maternal plasma lipids are involved in the pathogenesis of preterm birth

Yile Chen[1$], Bing He[1$], Yu Liu[1], Max T. Aung[2], Zaira Rosario-Pabón[3], Carmen M. Vélez-Vega[3], Akram Alshawabkeh[4], José F. Cordero[5], John D. Meeker[6], Lana X. Garmire[1 *]

[1]Department of Computational Medicine and Bioinformatics, University of Michigan, Ann Arbor, 48105, USA.

[2]Program on Reproductive Health and the Environment, Department of Obstetrics, Gynecology, and Reproductive Sciences, University of California, San Francisco, School of Medicine, San Francisco, CA, USA

[3]University of Puerto Rico Graduate School of Public Health, UPR Medical Sciences Campus, San Juan, Puerto Rico

[4]College of Engineering, Northeastern University, Boston, Massachusetts, United States

[5]Department of Epidemiology and Biostatistics, University of Georgia, Athens, Georgia, United States

[6]Department of Environmental and Health Sciences, School of Public Health, University of Michigan, Ann Arbor, 48109, USA.

[*] These authors contributed equally to the work.

[*] To whom correspondence should be addressed. Email address: lgarmire@med.umich.edu

Abstract

Background: Preterm birth is defined by the onset of labor at a gestational age shorter than 37 weeks and it can lead to premature birth and impose a threat to newborns' health. The Puerto Rico PROTECT cohort is a well-characterized prospective birth cohort that was designed to investigate environmental and social contributors to preterm birth in Puerto Rico, where preterm birth rates have been elevated in recent decades. To elucidate possible relationships between metabolites and preterm birth in this cohort, we conducted a nested case-control study to conduct untargeted metabolomic characterization of maternal plasma of 31 preterm birth women and 69 full-term labor controls at 24-28 gestational weeks.

Results: A total of 333 metabolites were identified and annotated with liquid chromatography/mass spectrometry. Subsequent weighted gene correlation network analysis shows the fatty acid and carene enriched module has a significant positive association (p-value=8e-04, FDR=0.006) with preterm birth. After controlling for potential clinical confounders, a total of 38 metabolites demonstrated significant changes uniquely associated with preterm birth, where 17 of them were preterm biomarkers. Among seven machine-learning classifiers, the application of random forest achieved the highly accurate and specific prediction (AUC = 0.92) for preterm birth in testing data, demonstrating their strong potential as biomarkers for preterm births. The 17 preterm biomarkers are involved in cell signaling, lipid metabolism, and lipid peroxidation functions. Additional modeling using only the 19 spontaneous preterm births (sPTB) and controls identifies 16 sPTB markers, with an AUC of 0.89 in testing data. Half of the sPTB overlaps with those markers for preterm births. Further causality analysis infers that suberic acid upregulates several fatty acids to promote preterm birth.

Conclusions: Altogether, this study demonstrates the involvement of lipids, particularly fatty acids, in the pathogenesis of preterm birth.

Keywords: preterm; metabolomics; lipid; metabolic pathway; biomarkers; network; fatty acid

Introduction

Preterm birth is defined as deliveries that occur prior to 37 weeks of gestation, and it is one of the leading causes of newborn mortality and morbidity [1]. We previously reported that the rates of preterm birth in Puerto Rico are among the highest observed worldwide, reaching 18% [2]. The Puerto Rico PROTECT cohort, herein referred to as the PROTECT cohort, was established to study the etiology of preterm birth and the risk factors associated with it. Factors such as higher maternal age [3], smoking history [4] and lower socioeconomic status, particularly as indicated by education level and income level [5] have been reported to be associated with adverse labor outcome [2]. Additionally, we conducted an environmental exposure study in PROTECT and found that phthalate metabolites were positively associated with preterm birth [6]. Endogenous metabolites derived from important biological processes (e.g. lipolysis, glycolysis) may provide critical insight into the etiology of antecedent mechanisms of preterm birth [7], therefore, we conducted a metabolomics study within the PROTECT cohort to establish a potential link between metabolites and preterm birth.

Metabolomics provides compositional and quantitative information about the state of an organism or cell at the macromolecular level [8]. Blood metabolomics has been used to identify biomarkers and potential molecular mechanisms for various diseases and conditions, such as aging [9], acute-on-chronic liver failure [10], hypertension and blood pressure progression [11]. Biomarkers of preterm birth have been discovered in the amniotic fluid, maternal urine/maternal blood, cervicovaginal fluid [7]. Decreased phosphocholine (PC) [12] and increased levels of acylglycerophosphoserines (PS), diacylglycerophosphoethanolamines (PE), phosphatidyinositol (PI), and phosphatiduglecerol (PG) were observed in maternal blood samples from women with preterm birth [13]. In a previous lipidomic analysis in the PROTECT cohort, we have also observed signals between maternal free fatty acids and phospholipids (plasmenyl-phosphatidylethanolamines) and spontaneous preterm birth [14]. We sought to expand on this body of

evidence and explore greater coverage of metabolic pathways and conducted this study to explore the potential roles of lipids in preterm birth.

The samples utilized in this study were maternal plasma collected in gestational weeks 24-28 from the women, who went on to experience preterm birth (N = 31) or full-term healthy deliveries (N = 69). Untargeted metabolomics LC-MS/MS assays were performed on these samples, followed by bioinformatics analysis. Our goals are the following: (1) identify metabolites and metabolomic pathways that are associated with preterm birth; (2) elucidate metabolomic processes that may have a causal relationship with preterm birth; (3) seek early gestational metabolomic biomarkers (week 24-28) that are predictive of preterm birth.

Materials and Methods

Study population

This study was conducted in an exploratory sample of the PROTECT cohort, which obtained its own institutional review boards (IRB) approval. This is a single-center study conducted in Puerto Rico. At the time of this study, the parent cohort consisted of 812 pregnant women, from which we randomly sampled 31 women who experienced preterm birth and 69 full-term controls for metabolomic analysis. Recruitment of the PROTECT cohort is ongoing and began in 2010. It's funded by the National Institute of Environmental Health Sciences Superfund Research Program. Participants were recruited in the first or second trimester of pregnancy (median 14 weeks gestation). Inclusion during early gestational age ranges allows for greater capacity to evaluate windows of vulnerability across pregnancy. Inclusion criteria for recruitment were: being 18-40 years of age; having residence in the Northern Karst aquifer region; disuse of oral contraceptives three months before pregnancy; disuse of *in vitro* fertilization; and lack of major health conditions or obstetrical complications in medical records. For preterm births, gestational ages <37

weeks were included. For controls, gestational ages between 39 weeks 0 days, and 40 weeks 6 days were included.

Pregnancy phenotypes

Medical records were used to determine birth outcomes. Gestational age in complete pregnancies was estimated using the American Congress of Gynecologists recommendations and previously described in greater detail [6,15,16]. The delivery less than 37 weeks gestation was defined to be preterm birth. Among preterm birth cases, we further disaggregated cases as spontaneous preterm birth cases if they had the presentation of premature rupture of membranes, spontaneous preterm birth, or both.

Sample preparation

Stored plasma samples, which were collected from the women between 24 and 28 weeks gestation and subsequently stored at -80C, were thawed on ice in preparation for analysis. Deproteinization was then performed by taking 100 µL of plasma combined with 400 µL 1:1:1 ratio of methanol, acetone, and water. Internal standards were also incorporated for metabolites recovery assessment and included: 5 µM of L-(D4)Thymine, L-[^{15}N] Anthranilic acid; and 20 µM of L-(^{15}N)$_2$ Tryptophan, Gibberellic acid, L-Epibrassinolide. Plasma samples were subsequently vortexed and centrifuged for 10 minutes at 15,000 x *g*. The supernatant of the centrifuged samples was transferred to a clean vial and dried using nitrogen gas. The dried samples were reconstituted to 50 µL.

Liquid chromatography-mass spectrometry untargeted metabolomics

The untargeted metabolomics analysis of all samples was randomly processed and assigned to liquid chromatography with a tandem mass spectrometry (LC-MS/MS) queue using a computerized algorithm. The reversed-phase chromatographic separation was performed on an Agilent 1290 Infinity II ultra-high performance liquid chromatography instrument (UHPLC) (Agilent Technologies, Inc., Santa Clara, CA USA) with the Waters Acquity BEH C18 column (Waters Corporation, Milford, MA). The temperature of

the column heater was maintained at 55ºC. The injection volume was 5 µL for all analyses. The lipid extract was injected onto a 1.7 µm particle diameter, 100× 2 mm id Waters Acquity BEH C18 column (Waters, Milford, MA) to separate the lipids. We used a linear gradient beginning with 98% Solvent A (water + 0.1% formic acid) and 2% Solvent B (methanol + 0.1% formic acid) to perform chromatographic elution. Solvent B was linearly increased to 98% over the first 22 minutes and was held at this level for 8 minutes. Thereafter, the composition was returned to the beginning and held for 3 minutes. The flow rate used for these experiments was 0.46 mL/min.

Mass spectrometry data acquisition for each sample was performed in both positive and negative ionization modes using an Agilent 6445 Q-TOF (AB Sciex, Concord, Canada). In positive ion mode runs, mobile phase A is 100% water that has 0.1% formic acid while mobile phase B is 100% methanol that has 0.1% formic acid. The formic acid is replaced with 0.1% (m/v) ammonium bicarbonate in negative ion mode runs. The column effluent was directed to the ESI source. For positive ionization mode, the voltage was set to 5500V. For negative ionization mode, the voltage was set to 4500V. For both modes, the declustering potential (DP) was set to 60 V and the source temperature was set to 450ºC. The curtain gas flow was 30. The nebulizer was 40. The heater gas was 45. The Q-TOF resolution according to specifications is greater than 45000 FWHM at 2722 m/z, mass accuracy was < 1ppm with in-line mass calibration, scan rate was ~118 scans per minute. Acquisition of MS/MS spectra was performed using the data-dependent acquisition (DDA) function of the Analyst TF software (AB Sciex, Concord, Canada). The software was set to the following parameters: dynamic background subtraction, charge monitoring to exclude multiply charged ions and isotopes, and dynamic exclusion of former target ions for 9 s.

Metabolite identification

The collected DDA MS/MS spectra data were analyzed using the Masshunter Qualitative Analysis Kit (AB Sciex, Concord, Canada). Using this kit, the “Find by Feature" algorithm is used to detect chromatographic peaks representative of metabolites. Between samples, feature alignment was performed using an in-house

written software package that matches features with identical mass and retention time between samples. In order to reduce gaps in chromatographic data, recursive feature identification was also performed by searching the data a second time with the list of aligned features using the "Find by Formula" algorithm in Agilent Masshunter Qualitative Analysis Software. Metabolites were putatively annotated using the mass spectral data annotation tool, Binner [17], to reduce contaminants, artifacts, and degeneracies. An annotated metabolite list was searched against an in-house library of 800 known metabolite standards which had been previously analyzed under identical LC-MS conditions. MS/MS spectra for metabolites not identified by standards were searched in the Metlin (Agilent Metlin B.08.00) or NIST 17. Metabolites not identified by library standards or MS/MS spectra were searched in the Metlin database (http://metlin.scripps.edu) and Human Metabolome Database (HMDB; http://www.hmdb.ca).

Metabolomics data preprocessing and quality check

Samples were assayed in a single batch. Pooled quality control (QC) samples were prepared by pooling equal volumes of each sample. The pooled QC samples were injected at the beginning and the end of each analysis and after every 10 sample injections to provide a measurement of the system's stability and performance. The PCA plots of cases, controls, and pool QC samples are shown in Supplementary Fig. S4. A total of 333 metabolites species were detected using the DDA MS/MS spectra data collected either in positive ion mode or negative ion mode. Missing value imputation was performed using the K-nearest neighbors (KNN) method [18]. Log-transformation and quantile normalization [19] was applied to the data, prior to the other downstream analysis. For quality check, partial least squares-discriminant analysis (PLS-DA) was applied on the 100 samples using all identified metabolites.

Source of variation analysis and data screen

The metabolomics dataset of maternal plasma consists of 333 metabolites, including 167 metabolites in the negative mode and 166 metabolites in the positive mode. To eliminate confounders that are not truly related to preterm birth, we conducted a preliminary screen according to the source of variation (SOV) analysis,

which helps to discover the contributions of each clinical/physiological factor to the metabolomics variation. The metabolites with an F statistic of preterm/control > 1 were screened before other analyses, meaning that they had a regression sum of squares larger than the error sum of squares. All 333 metabolites passed this screening process.

Differential metabolomics species identification

To remove potential confounding effects, we fit a linear model for each metabolite over preterm status while adjusting for *a priori* phenotypic variables via the R *limma* package [20]. Adjusted phenotypic variables include gestational age in weeks, smoking status, alcohol consumption, baby length, baby gender (fetal sex), LGA/SGA (large/small for gestational age), maternal age, income, and pre-pregnancy BMI. Large for gestational age (LGA) describe infants that are born with an abnormally high weight, specifically in the 90th percentile or above, compared to other babies of the same developmental age. Small for gestational age (SGA) describes infants whose weight is less than the 10th percentile for gestational age. Metabolites with p-values < 0.05 were selected as statistically significant in association with preterm birth.

Weighted gene co-expression network analysis

For the weighted gene co-expression network analysis (WGCNA) analysis, all metabolites were analyzed together [21]. The smallest soft threshold with an adjusted $R^2 > 0.8$ was 4, and hence it was chosen to calculate the adjacency score between any 4 metabolites within a sample set. Following that, the topological overlap value between these 4 metabolites was computed from this adjacency score and the corresponding connectivity value [22]. The topological overlap value is converted to a distance value by minus it from 1 and producing a pairwise metabolites distance matrix. This distance matrix was then used to cluster the metabolites using hierarchical clustering with dendrogram, where modules were identified. As a result, we kept the metabolites with their topological overlap score larger than 0.5 in each module. For the integrated WGCNA analysis using both preterm and healthy samples, we used a soft threshold (power) of 8 as

suggested by the WGCNA estimation. We setted minModuleSize 10, mergeCutHeight 0.25, deepSplit 2 and verbose 3 for the WGCNA analysis.

The model of classification

We first further screened the differentiated metabolites with mutual information (MI) greater than 0.5 and then utilized the *Lilikoi* package [23] to determine the best machine learning model for classifying preterm and control samples using selected metabolites. Seven algorithms were compared in this step: recursive partitioning and regression trees (RPART), partition around medoids (PAM), gradient boosting (GBM), logistic regression with elastic net regularization (LOG), random forest (RF), support vector machine (SVM), and linear discriminant analysis (LDA). The samples were randomly split into 80/20 ratio for training data vs. testing data. The best method was determined on the training set using 10-fold cross-validation, by metrics F-statistics and balanced accuracy. We applied the same process above on the subset of 19 spontaneous preterm birth (sPTB) cases and controls.

The mapping of metabolite-related pathway and phenotype

We used the query lipid as the input to map metabolites to pathways from HMDB, PubChem, and KEGG in *Lilikoi [23,24]*. These metabolite-pathway interactions were then used for further pathways analysis. Pathway dysregulation scores (PDS), a metric representing the degree of dysregulation at the pathway level, were calculated through the Pathifier R package to determine the dysregulation level of the pathway [25].

Causality analysis

We sorted metabolomics data and clinical features into time series by the gestational ages of patients. Then we performed the Granger causality test to identify potential causality relationships between metabolites and preterm birth using the lmtest R package (version 0.9-37). The threshold of the p-value is set to 0.01 for significant causality interaction.

Results

Study overview

The demographic and major clinical characteristics of the subjects in the PROTECT cohort study is shown in Table 1. Except for the fact that individuals with preterm deliveries have significantly shorter gestational ages than healthy pregnant women (mean gestational age 39.20 weeks vs. 34.69 weeks, p-value = 1.28e-13), other characteristics of cases and controls are comparable across all categories. Women with preterm birth show the trend of higher mean BMI values than controls (27.51 vs. 25.55) , however this is not statistically significant (p=0.165). We also investigated the correlations among phenotypic factors (Fig. 1A). Income is positively correlated with preterm birth in weeks (PCC_{Income} = 0.205, p-value<0.05), confirming the social-economic association with preterm birth [26]. Maternal age shows the tendency of negative correlation with preterm (PCC_{Age} = -0.181, p<0.1).

A total of 333 lipid metabolites were identified by LC/MS in maternal plasma. Partial least squares-discriminant analysis (PLS-DA) plot of the 100 samples using all identified lipid metabolites shows that preterm samples are well separated from healthy controls using the first two components (Fig. 1B). To examine the degree of confounding from other variables, a source of variation (SOV) analysis was carried out (Fig. 1C). Preterm birth is ranked the first for the F statistics, followed by variables BMI, income, maternal age, alcohol consumption, smoking, and SGA which all have F statistics bigger than 1. To further identify the relationships between phenotypic factors and metabolites, correlations between clinical factors and metabolites were calculated (Fig. 1D) and then subject to hierarchical clustering (using Euclidean distance as the distance metric). Three Clusters of metabolites are identified with sizes of 230, 36, and 67. Cluster 3 is significantly enriched in fatty acids (FAs) (Fisher's p-value = 5.24e-4, FDR=0.02, odds ratio = 2.12), and FAs are generally lower in preterm samples. They have a striking pattern of negative associations

with preterm birth. Moreover, FAs also have overall negative associations with age, income, and alcohol use, suggesting the biological, social-economical, and behavioral effects are intertwined at the metabolomic level. The other two clusters do not have enrichment in specific metabolite functional groups.

Correlation network analysis of metabolomics related to preterm birth

To further elucidate the relationships between metabolomics and preterm birth, we next performed the weighted gene correlation network analysis (WGCNA) method on the 333 metabolites [21]. WGCNA analysis yields 7 modules (Fig. 2A). Among these modules, only the turquoise-colored module shows a significant positive association (Fisher's Exact Test, p-value=8e-04, FDR=0.006) with preterm birth (Fig. 2A-B). This module is enriched with FAs (Fisher's Exact Test, p-value=3.85e-05, FDR=4.24e-04) and carene (CAR) (Fisher's Exact Test, p-value=2.53e-03, FDR=0.028). This FA/CAR enriched module also shows a significant negative association (p-value=0.002, FDR=0.022) with gestational age (GestAge) (Fig. 2B). These results, together with the previous metabolite-phenotype analysis (Fig. 1C), demonstrate that FAs in the mothers who gave birth prematurely not only have higher levels but also tighter correlations (through regulations). To examine the module difference between cases and controls more closely, we further conducted the WGCNA on the two groups separately. Three modules have significantly overlapping metabolites in the case and control groups (Supplementary Fig. S1. 2A and 2B), respectively. Interestingly, the FA enriched modules in cases (A2) and controls (B2) have the most significant overlap (p-value=6.76e-18, FDR=6.084e-17) (Supplementary Fig. S1. 2C). However, we did not find that the density of FA-enriched modules was higher in preterm cases compared to that in control (Supplementary Fig. S1. 2D).

Differentiated metabolites and their mapped pathways

We next conducted differential metabolite analysis between cases and controls, using *limma* package [20] allowing for phenotypic variable adjustment. As a result, 38 metabolites are significantly different (p-value <0.05) between preterm and control samples exclusively, and are not associated with other confounders (Fig. 3A). The log fold changes (logFC) of the differentiated metabolites ranged from -0.87 to 0.68

(Supplementary Table S1). Among them, 21 metabolites are up-regulated and 17 metabolites are down-regulated in preterm samples (Fig. 3B). The majority of these metabolites are unsaturated fatty acids.

To further explore the functions of these metabolites, we mapped the 333 metabolites to pathways and conducted pathway enrichment analysis, using the *Lilikoi* R package [18,23]. These pathways are from KEGG, HMDB, Metlin and PubChem databases. 240 out of 333 metabolites are successfully mapped by at least one database, with assigned memberships to 38 pathways. Among the 38 differential metabolites, 33 of them are involved in 5 pathways that show significant alterations in pathway dysregulation scores, a metric representing the degree of dysregulation at the pathway level [25]. These pathways share a lot of lipids and are interrelated: Lipid metabolism, cell signaling, lipid transport, fatty acid metabolism, and lipid peroxidation. The bipartite plot illustrated the relationships between the differentiated metabolites and their corresponding differential pathways (Fig. 3C).

Metabolomics based preterm biomarker model

Another important application of metabolomics analysis is to screen for diagnostic biomarkers for diseases. For this purpose, we split samples with 80/20 ratio into training and testing data. We further selected 17 metabolites out of the 38 differentiated ones using mutual information score of 0.5 as the threshold. We compared the performance of seven machine learning algorithms in the *Lilikoi* R package, including recursive partitioning and regression trees (RPART), partition around medoids (PAM), gradient boosting (GBM), logistic regression with elastic net regularization (LOG), random forest (RF), support vector machine (SVM), and linear discriminant analysis (LDA). We used the area under the ROC curve (AUC), F1 statistic, and balanced accuracy to evaluate the models. Among all classification methods, RF yields the highest balanced accuracy statistic (1.0) in the training dataset (Fig. 4A), so we selected it as the winning model to show the predictive performance on the remaining testing dataset. The overall accuracy for RF on the testing data is 0.92 for the AUC, 0.5 for the F1 statistic, and 0.67 for the balanced accuracy (Fig. 4C). Next, we tested if the biomarkers are specific to preterm birth rather than other clinical confounders. We

used the 17-feature RF classification model built for preterm birth to predict its classification performance over other terms including LGA, BMI, and maternal age, using the same testing data set. The AUC on LGA, BMI, and maternal age are 0.2, 0.09, and 0.17 respectively in the precision-recall curves (Fig 4D). This confirms the specificity of the 17-biomarker model for preterm birth. Several fatty acids show top importance scores in the model: FA(17:1) (1st, importance score = 7.32 out of 100); FA(24:6) (2nd, 7.02); FA14:2 (3rd, 6.98). Hexanoylcarnitine is also a top important metabolite (5th, 6.6), involved in fatty acid oxidation. It has been reported to be significantly higher in preterm birth [27].

Predicted causality interactions among metabolites and preterm birth

We used the Granger causality test [28] to infer significant causality interactions (p-value < 0.01) between the 17 metabolites and the binary preterm outcome. As shown in Fig. 5, up-regulated hexanoylcarnitine (logFC = 0.472), CAR(18:2) (logFC = 0.375), CAR(20:2) (logFC = 0.280), FA(14:1(Ke)) (logFC = 0.407), FA(14:2) (logFC = 0.492), FA(17:1) (logFC = 0.402), and down-regulated behenic acid (logFC =-0.191), pimelic acid (logFC =-0.357), suberic acid (logFC =-0.224), glycocholic acid (logFC =-0.867), and PC(33:4) (logFC =-0.332) are predicted as direct casual metabolites of preterm birth. The causality test also predicts the causality interaction from FA(17:1) to pimelic acid, which is synthesized from fatty acid [29]. Interestingly, down-regulated suberic acid (logFC = -0.224) is predicted to be the direct cause of up-regulated FA(22:4) (logFC = 0.332), FA(20:2) (logFC = 0.282), FA(22:2) (logFC = 0.221), FA(14:0(Ke)) (logFC = 0.434), FA(14:1(Ke)) (logFC = 0.407), and FA(14:2) (logFC = 0.492). A previous study shows that suberic acid is present in the urine of patients with fatty acid oxidation disorders, indicating the correlation between suberic acid and the metabolism of fatty acids [30].

Prediction model for spontaneous preterm birth (sPTB)

The 31 preterm samples include 19 sPTB cases and 12 samples from other conditions (eg. preeclampsia). To further investigate the association between metabolites and sPTB, we analyzed the cases with sPTB separately (Case = 19; Control = 75). We conducted differential analysis between sPTB and controls, and

identified 53 metabolites with p-values < 0.05, 33 of which also appeared in the previous 38 metabolites significantly different in preterms vs. controls (Supplementary Fig. S2A). For the 33 metabolites, the differential patterns are consistent in both preterm birth and spontaneous preterm birth, with the fold changes being more extreme in sPTB (Supplementary Fig. S2B).

Using the same procedures as in the previous metabolomics-based preterm biomarker model, we identified 16 out of the 55 metabolites as markers for sPTB. Half of these 16 markers, including FA(24:6), FA(16:3), FA(17:1), FA(14:2), FA(19:1), FA(14:0(Ke)), FA(14:1) and heptadecanoic acid, are also among the previously identified 17 metabolite markers for preterm birth. We further investigated whether the identified markers can serve as good predictors of sPTB (Supplementary Fig. S3). RF method again presents the best performance in the training data and achieves an AUC of 0.89 in the testing data. In the metabolite marker importance ranking, previously 5th-ranked hexanoylcarnitine appears again in the top metabolites (5th, 7.56). In summary, many preterm markers are also robust sPTB signatures.

Discussion

Preterm birth is one of the leading causes of newborn mortality and morbidity [1]. To improve our understanding of preterm birth, we conducted a metabolomics analysis of maternal blood in the PROTECT cohort of preterm birth patients and healthy controls.

The importance of fatty acids in preterm birth is highlighted by bioinformatics analysis in various aspects. First, correlation network analysis of metabolomics reveals deregulated lipid modules that may contribute to preterm birth (Fig. 2). The FA/CAR enriched module is enriched with several fatty acids including two essential fatty acids, i.e. alpha-linolenic acid and linoleic acid (omega-6 fatty acid), and a class of saturated fatty acids (SFAs: heptadecanoic acid, palmitic acid). Second, fatty acids show high importance scores in machine learning models for either preterm birth or spontaneous preterm birth. Other studies have also found excessive free fatty acids detected in the preterm cases of maternal circulation, linking them to

inflammation [31] the main cause of preterm birth [27]. In fact, a higher omega-6 to omega-3 fatty acid ratio would increase pro-inflammatory eicosanoid production [32,33] and it was associated with shorter gestation duration for overweight/obese women [34]. Another study on underweight and obese women with spontaneous preterm birth identified a higher concentration of omega-6 and omega-3 fatty acids in their mid-gestation serum samples [35]. Confirming our discovery, a recent complementary lipidomics study within the PROTECT cohort also observed that mono- and poly-unsaturated free fatty acids (FFA 20:1, FFA 20:1, FFA 18:1) were associated with a higher risk of spontaneous preterm birth [14]. We have also found complimentary evidence in the LIFECODES cohort of positive associations between spontaneous preterm birth and eicosanoids, which are secondary metabolites of poly-unsaturated fatty acid parent compounds such as arachidonic acid [36]. Besides fatty acids, two phosphatidylcholine (PC(18:0/16:0), PC(33:4)) were also selected by the biomarker model for preterm birth. These two metabolites have lower levels in preterm births. PCs are the main structure of cell membranes and play an important role in maintaining membrane stability and reducing inflammation [37]. Consistent with this, one recent study also found a class of PC significantly lower in preterm births [38].

Interestingly, the causality analysis shows the causal effect of decreased suberic acid for the excessive fatty acids. This is consistent with a previous finding that suberic acid is related to fatty acid disorders [30]. Suberic acid, also called octanedioic acid, is a dicarboxylic acid, which can be produced from fatty acids [39]. The production from fatty acids to dicarboxylic acids is catalyzed by cytochrome P450 (CYP) 4 F/A (CYP4F/A) enzymes [39,40]. The accumulation of fatty acids and reduction of suberic acid in preterm maternal blood samples (Fig 5) suggest that CYP4F/A enzymes, the enzyme catalyzing this conversion, have reduced activities in preterm delivery. Polymorphisms in CYP4F/A genes, which impair enzyme functions, previously showed associations with preterm birth [41]. Thus, we speculate that polymorphisms or other forms of deactivation of CYP4F/A genes may play a role in preterm births.

Changes in these lipids collectively suggest that lipid metabolism may contribute to the pathogenesis of preterm birth (Fig 6). Indeed, several related pathways including lipid metabolism, fatty acid metabolism,

and lipid peroxidation pathways are all enriched in the preterm cases (Fig 3C). These pathways were discussed frequently in many previous preterm birth analyses [38,42,43]. UFAs, shown to be excessive in preterm samples of this dataset, are more likely to undergo lipid peroxidation[44]. UFAs and the evident lipid peroxidation process could lead to oxidative stress, which was reportedly related to preterm birth through regulating ripening cervical, uterus contraction, and membrane rupture [42]. and accelerated lipid peroxidation is found in prematurity [45].

A few caveats of this study should also be mentioned. First, subjects in this study do not have specific dietary records, thus potential confounding from diets cannot be investigated. The metabolites are measured from maternal blood, therefore any biological mechanisms discussed here are inferred systematically rather than being directly measurable from relevant tissues (eg. placenta). Additionally, despite developing a potential biomarker panel from a classification model, these candidates are suggestive and not quantitatively validated yet. We plan to validate them in other independent cohorts in the future. Nonetheless, this study provides strong evidence of the involvement of a class of saturated and unsaturated FAs and PCs in preterm births, mediated by perturbation in biological functions including cell signaling and lipid peroxidation.

Availability of source code and requirements

Project name: Maternal lipids in the pathogenesis of preterm birth

Project home page: https://github.com/lanagarmire/pretermBirth_metabolomics

Operating system(s): Windows and Linux

Programming language: R

Data Availability

This data is available at the NIH Common Fund's National Metabolomics Data Repository (NMDR) website, the Metabolomics Workbench, https://www.metabolomicsworkbench.org where it has been assigned Project ID (PR001155). The data can be accessed directly via it's Project DOI: (http://dx.doi.org/10.21228/M8DH5P). This work is supported by NIH grant U2C-DK119886. Other data further supporting this work are openly available in the *GigaScience* repository, GigaDB [46].

Abbreviations

LDA: linear discriminant analysis; RF: random forest; LOG: elastic net; GBM: gradient boosting; SVM: support vector machine; RPART: classification tree; PC: phosphocholine; PS: acylglycerophosphoserines; PE: diacylglycerophosphoethanolamines; PI: phosphatidyinositol; PG: phosphatiduglecerol; FA: fatty acid; CAR: carene; CYP4F/A: cytochrome P450 (CYP) 4 F/A; AUC: area under the ROC curve; WGCNA: weighted gene correlation network analysis; SOV: source of variation.

Competing Interests

The authors declare that they have no competing interests.

Author's contribution

YC and BH conducted the bioinformatics analysis, modified code provided by YL. MA provided writing material. JDM designed the study, obtained funding, supervised the metabolomics assays, and critically reviewed early drafts of the paper. LXG supervised the analysis. YC, BH and LXG wrote the manuscript. All authors have read and revised the manuscript.

Funding

This study was supported by the Superfund Research Program of the National Institute of Environmental Health Sciences, National Institutes of Health (grants P42ES017198). Additional support was provided from NIEHS grant numbers P50ES026049, R01ES032203, and P30ES017885 and the Environmental influences on Child Health Outcomes (ECHO) program grant number UH3OD023251. LXG is supported by grants K01ES025434 awarded by NIEHS through funds provided by the trans-NIH Big Data to Knowledge (BD2K) initiative (www.bd2k.nih.gov), R01 LM012373 and LM012907 awarded by NLM, R01 HD084633 (LXG and SS) awarded by NICHD.

Acknowledgment

We thank the nurses and research staff who participated in cohort recruitment and follow up, as well as the Federally Qualified Health Centers (FQHC) and clinics in Puerto Rico who facilitated participant recruitment, including Morovis Community Health Center (FQHC), Prymed: Ciales Community Health Center (FQHC), Camuy Health Services, Inc. (FQHC), and the Delta OBGyn(Prenatal Clinic). We thank the BRCF Metabolomics core of the University of Michigan for providing the assays and helping upload the metabolomics data to the public repository.

Tables

Table 1. Demographic and clinical characteristics for subjects in this study.

Figure Legends

Figure 1. (A) Correlation matrix of the 10 phenotypic variables on the 100 samples (69 controls vs 31 preterm cases). (B) Partial least squares-discriminant analysis (PLS-DA) plot of the 100 samples using 333 metabolites. (C) Source of variation (SOV) analysis using 100 samples. 333 metabolites are used in the ANOVA model. (D) Heatmap of correlations between 333 metabolites and 11 confounding factors. The rows represent the clinical factors, and the columns represent metabolites (Point-Biserial Correlation for

continuous and binary covariates; Pearson Correlation for continuous covariates; Spearman Correlation for continuous and ordinal covariates).

Figure 2. WGCNA network in all samples. (A) WGCNA network modules of metabolomics data from both preterm and control samples. Each node represents a lipid. Node color represents a module. (B) Module-trait associations.

Figure 3. Metabolites show significantly different levels in preterm and control samples. (A) Heatmap of the 38 metabolites with a significant difference exclusively between preterm and control samples (p-value <0.05). (B) Barplots on the averaged normalized intensities in cases vs controls. (C) Bipartite graph of the significantly differentiated metabolites and the significantly altered metabolic pathways they are associated with. Five pathways with a significant difference between preterm and control samples (p-value <0.05) and 33 significantly differentiated metabolites engaged in these pathways are shown. Elliptical nodes: metabolites. Rectangular nodes: pathways from HMDB, PubChem, and KEGG databases. Node color: Red, up-regulated; Blue, Down-regulated. Node size: the absolute value of log fold change (logFC).

Figure 4. Classification model for preterm birth. (A) Comparison of seven classification models using 17 metabolites on the hold-out testing. The data set was randomly split into training data (80%) and testing data (20%) 10 times. The average value and standard error of the 10 repeats are shown for three performance metrics of the area under the ROC curve (AUC), F1 statistic, and balanced accuracy. The winning method RF in training data (left) was then applied to the testing data (right). (B) The heatmap of correlation coefficients between the 17 metabolites and clinical variables. (C) The precision-recall curves of the RF model from (A) on classifying preterm, LGA (large for gestational age), Income, and Maternal Age (>=35 yrs or not) respectively, using the same set of testing data as in (A). (D) Normalized variable importance scores for the 17 lipid markers in the RF model. The normalization is done on R by making the sum of importance scores to be 100.

Figure 5. Predicted significant (p-value < 0.01) causality interactions between the 17 metabolites and preterm birth. Arrow indicates the causality interaction. Blue and red nodes are down and up-regulated metabolites, while the center one is preterm.

Figure 6. A proposed model of metabolites changes affecting preterm birth.

Supplementary materials

Supplementary Figure S1. (A-B) WGCNA network in preterm births (A) and healthy controls (B), respectively. Each node represents a metabolite, whose size is proportional to the node connectivity value in a WGCNA network. (C) The overlap between modules of networks in control and preterm samples. (D) Detailed information on overlapping module density was discovered in (C). (E) Bar plot of the connectivity scores of the 17 up-regulated metabolites.

Supplementary Figure S2. Metabolites show significantly different levels in sPTB and control samples. (A) Heatmap of the 55 metabolites with a significant difference exclusively between sPTB and control samples (p-value <0.05). (B) Barplots on the averaged normalized intensities in cases vs controls.

Supplementary Figure S3. Classification model for sPTB. (A) Comparison of seven classification models using 16 metabolites on the hold-out testing. The data set was randomly split into training data (80%) and testing data (20%) 10 times. The average value and standard error of the 10 repeats are shown for three performance metrics of the area under the ROC curve (AUC), F1 statistic, and balanced accuracy. The winning method RF in training data (left) was then applied to the testing data (right). (B) The heatmap of correlation coefficients between the 16 metabolites and clinical variables. (C) The precision-recall curves of the RF model from (A) on classifying preterm, LGA (large for gestational age), income, and maternal Age (>=35 yrs or not) respectively, using the same set of testing data as in (A). (D) Normalized variable

importance scores for the 16 lipid markers in the RF model. The normalization is done by making the sum of importance scores as 100.

Supplementary Figure S4. PCA plots for the QC of the metabolomics data in positive and negative modes.

Supplementary Table S1. Fold change values of the 38 metabolites that are significantly different between preterm and control samples

References

1. Callaghan WM, MacDorman MF, Rasmussen SA, Qin C, Lackritz EM. The contribution of preterm birth to infant mortality rates in the United States. *Pediatrics*. 118:1566–732006;

2. Ferguson KK, Rosario Z, McElrath TF, Vélez Vega C, Cordero JF, Alshawabkeh A, et al.. Demographic risk factors for adverse birth outcomes in Puerto Rico in the PROTECT cohort. *PLoS One*. 14:e02177702019;

3. Cleary-Goldman J, Malone FD, Vidaver J, Ball RH, Nyberg DA, Comstock CH, et al.. Impact of maternal age on obstetric outcome. *Obstet Gynecol*. 105:983–902005;

4. Shah NR, Bracken MB. A systematic review and meta-analysis of prospective studies on the association between maternal cigarette smoking and preterm delivery. *Am J Obstet Gynecol*. 182:465–722000;

5. Thayamballi N, Habiba S, Laribi O, Ebisu K. Impact of Maternal Demographic and Socioeconomic Factors on the Association Between Particulate Matter and Adverse Birth Outcomes: a Systematic Review and Meta-analysis. Journal of Racial and Ethnic Health Disparities.

6. Ferguson KK, Rosen EM, Rosario Z, Feric Z, Calafat AM, McElrath TF, et al.. Environmental phthalate exposure and preterm birth in the PROTECT birth cohort. Environment International.

7. Gil AM, Duarte D. Biofluid Metabolomics in Preterm Birth Research. *Reprod Sci*. 25:967–772018;

8. Li S, Todor A, Luo R. Blood transcriptomics and metabolomics for personalized medicine. *Comput Struct Biotechnol J*. 14:1–72016;

9. Kondoh H, Kameda M, Yanagida M. Whole Blood Metabolomics in Aging Research. *Int J Mol Sci*. 2020; doi: 10.3390/ijms22010175.

10. Moreau R, Clària J, Aguilar F, Fenaille F, Lozano JJ, Junot C, et al.. Blood metabolomics uncovers inflammation-associated mitochondrial dysfunction as a potential mechanism underlying ACLF. *J Hepatol*. 72:688–7012020;

11. Lin Y-T, Salihovic S, Fall T, Hammar U, Ingelsson E, Ärnlöv J, et al.. Global Plasma Metabolomics to Identify Potential Biomarkers of Blood Pressure Progression. *Arterioscler Thromb Vasc Biol*. 40:e227–372020;

12. Pinto J, Maciel E, Melo TS, Domingues MRM, Galhano E, Pita C, et al.. Maternal plasma phospholipids are altered in trisomy 21 cases and prior to preeclampsia and preterm outcomes. *Rapid Commun Mass Spectrom*. 28:1635–82014;

13. Virgiliou C, Gika HG, Witting M, Bletsou AA, Athanasiadis A, Zafrakas M, et al.. Amniotic Fluid and Maternal Serum Metabolic Signatures in the Second Trimester Associated with Preterm Delivery. *J Proteome Res*. 16:898–9102017;

14. Aung MT, Ashrap P, Watkins DJ, Mukherjee B, Rosario Z, Vélez-Vega CM, et al.. Maternal lipidomic signatures in relation to spontaneous preterm birth and large-for-gestational age neonates. *Sci Rep*. 11:81152021;

15. Committee on Obstetric Practice, the American Institute of Ultrasound in Medicine, and the Society for Maternal-Fetal Medicine. Committee Opinion No 700: Methods for Estimating the Due Date. *Obstet Gynecol*. 129:e150–42017;

16. Aker AM, Ferguson KK, Rosario ZY, Mukherjee B, Alshawabkeh AN, Cordero JF, et al.. The associations between prenatal exposure to triclocarban, phenols and parabens with gestational age and birth weight in northern Puerto Rico. *Environ Res*. 169:41–512019;

17. Kachman M, Habra H, Duren W, Wigginton J, Sajjakulnukit P, Michailidis G, et al.. Deep annotation of untargeted LC-MS metabolomics data with Binner. *Bioinformatics*. 36:1801–62020;

18. Alakwaa FM, Chaudhary K, Garmire LX. Deep Learning Accurately Predicts Estrogen Receptor Status in Breast Cancer Metabolomics Data. *J Proteome Res*. 17:337–472018;

19. De Livera AM, Dias DA, De Souza D, Rupasinghe T, Pyke J, Tull D, et al.. Normalizing and integrating metabolomics data. *Anal Chem*. 84:10768–762012;

20. Ritchie ME, Phipson B, Wu D, Hu Y, Law CW, Shi W, et al.. limma powers differential expression analyses for RNA-sequencing and microarray studies. *Nucleic Acids Res*. 43:e472015;

21. Zhang B, Horvath S. A general framework for weighted gene co-expression network analysis. *Stat Appl Genet Mol Biol*. 4:Article172005;

22. Langfelder P, Horvath S. WGCNA: an R package for weighted correlation network analysis. *BMC Bioinformatics*. 9:5592008;

23. Fang X, Liu Y, Ren Z, Du Y, Huang Q, Garmire LX. Lilikoi V2.0: a deep learning-enabled, personalized pathway-based R package for diagnosis and prognosis predictions using metabolomics data. *Gigascience*. 2021; doi: 10.1093/gigascience/giaa162.

24. Al-Akwaa FM, Yunits B, Huang S, Alhajaji H, Garmire LX. Lilikoi: an R package for personalized pathway-based classification modeling using metabolomics data. *Gigascience*. 2018; doi: 10.1093/gigascience/giy136.

25. Drier Y, Sheffer M, Domany E. Pathway-based personalized analysis of cancer. *Proc Natl Acad Sci U S A*. 110:6388–932013;

26. Kramer MS, Goulet L, Lydon J, Seguin L, McNamara H, Dassa C, et al.. Socio-economic disparities in preterm birth: causal pathways and mechanisms. Paediatric and Perinatal Epidemiology.

27. Elshenawy S, Pinney SE, Stuart T, Doulias P-T, Zura G, Parry S, et al.. The Metabolomic Signature

of the Placenta in Spontaneous Preterm Birth. *Int J Mol Sci*. 2020; doi: 10.3390/ijms21031043.

28. Geweke J. Causality, exogeneity, and inference. Advances in Econometrics.

29. Manandhar M, Cronan JE. Pimelic acid, the first precursor of the Bacillus subtilis biotin synthesis pathway, exists as the free acid and is assembled by fatty acid synthesis. *Mol Microbiol*. 104:595–6072017;

30. Hagen T, Korson MS, Sakamoto M, Evans JE. A GC/MS/MS screening method for multiple organic acidemias from urine specimens. *Clin Chim Acta*. 283:77–881999;

31. Calder PC. Fatty acids and inflammation: the cutting edge between food and pharma. *Eur J Pharmacol*. 668 Suppl 1:S50–82011;

32. Coletta JM, Bell SJ, Roman AS. Omega-3 fatty acids and pregnancy. *Rev Obstet Gynecol*. MedReviews, LLC; 3:1632010;

33. Simopoulos AP. The importance of the ratio of omega-6/omega-3 essential fatty acids. *Biomed Pharmacother*. 56:365–792002;

34. Penfield-Cyr A, Monthe-Dreze C, Smid MC, Sen S. Maternal BMI, Mid-pregnancy Fatty Acid Concentrations,and Perinatal Outcomes. *Clin Ther*. 40:1659–67.e12018;

35. Borkowski K, Newman JW, Aghaeepour N, Mayo JA, Blazenović I, Fiehn O, et al.. Mid-gestation serum lipidomic profile associations with spontaneous preterm birth are influenced by body mass index. *PLoS One*. 15:e02391152020;

36. Aung MT, Yu Y, Ferguson KK, Cantonwine DE, Zeng L, McElrath TF, et al.. Prediction and associations of preterm birth and its subtypes with eicosanoid enzymatic pathways and inflammatory markers. *Sci Rep*. 9:170492019;

37. van Meer G, Voelker DR, Feigenson GW. Membrane lipids: where they are and how they behave. *Nat Rev Mol Cell Biol*. Springer Science and Business Media LLC; 9:112–242008;

38. Morillon A-C, Yakkundi S, Thomas G, Gethings LA, Langridge JI, Baker PN, et al.. Association between phospholipid metabolism in plasma and spontaneous preterm birth: a discovery lipidomic analysis in the cork pregnancy cohort. *Metabolomics*. 16:192020;

39. Dhar M, Sepkovic DW, Hirani V, Magnusson RP, Lasker JM. Omega oxidation of 3-hydroxy fatty acids by the human CYP4F gene subfamily enzyme CYP4F11. *J Lipid Res*. 49:612–242008;

40. Wanders RJA, Komen J, Kemp S. Fatty acid omega-oxidation as a rescue pathway for fatty acid oxidation disorders in humans. *FEBS J*. 278:182–942011;

41. Guleria K. Assessment of toxicogenomic risk factors in etiology of preterm delivery. *Reprod Syst Sex Disord*. OMICS Publishing Group; 2014; doi: 10.4172/2161-038x.1000129.

42. Menon R. Oxidative stress damage as a detrimental factor in preterm birth pathology. *Front Immunol*. 5:5672014;

43. Cappelletti M, Della Bella S, Ferrazzi E, Mavilio D, Divanovic S. Inflammation and preterm birth. *J Leukoc Biol*. 99:67–782016;

44. Li J, Lu YP, Reichetzeder C, Kalk P, Kleuser B, Adamski J, et al.. Maternal PCaaC38:6 is Associated With Preterm Birth - a Risk Factor for Early and Late Adverse Outcome of the Offspring. Kidney and Blood Pressure Research.

45. Chakravartya S, Sontakkeb A. A correlation of antioxidants and lipid peroxidation between maternal and cord blood in full term and preterm deliveries. *Curr Pediatr Res*. 16:167–742012;

46. Chen Y; He B; Liu Y; Aung MT; Rosario-Pabón Z; Vélez-Vega CM; Alshawabkeh A; Cordero JF; Meeker JD; Garmire LX: Supporting data for "Maternal plasma lipids are involved in the pathogenesis of preterm birth" GigaScience Database. 2022. http://dx.doi.org/10.5524/100965.

 Click here to access/download;Table;Table1.pdf

Table 1. Demographic and clinical characteristics in case and control groups.

	Control (n=69)<br>Mean (SD)	Case (n=31)<br>Mean (SD)	P-value*
Maternal age, years	27.07 (5.91)	24.84 (5.10)	0.058
BMI, kg/m^2	25.55 (5.25)	27.51 (6.92)	0.165
Gestational Age, weeks	39.20 (0.98)	34.69 (2.08)	1.28e-13
Income<br>1= less than $4,999<br>2= $5,000-$9,999<br>3= $10,000-$19,999<br>4= $20,000-$29,999<br>5= $30,000-$39,999<br>6= $40,000-$49,999<br>7= $50,000-$74,999<br>8= $75,000-$99,999<br>9= $100,000-$199,999	3.87 (2.12)	2.87 (2.22)	0.039
Baby Gender<br>Female<br>Male	 35<br>34	 14<br>17	0.669
Smoker<br>Yes<br>No	 12<br>57	 2<br>29	0.215
Alcohol<br>no use during pregnancy<br>drank before pregnancy<br>drank during pregnancy<br>Unknown	 32<br>32<br>4<br>1	 19<br>9<br>2<br>1	0.294
SGA (small-for-gestational-age)<br>No<br>Yes<br>Unknown	 58<br>10<br>1	 25<br>6<br>0	0.567

*t test for continuous variable and Fisher's exact test for count data.

Click here to access/download;Figure;Fig1.pdf

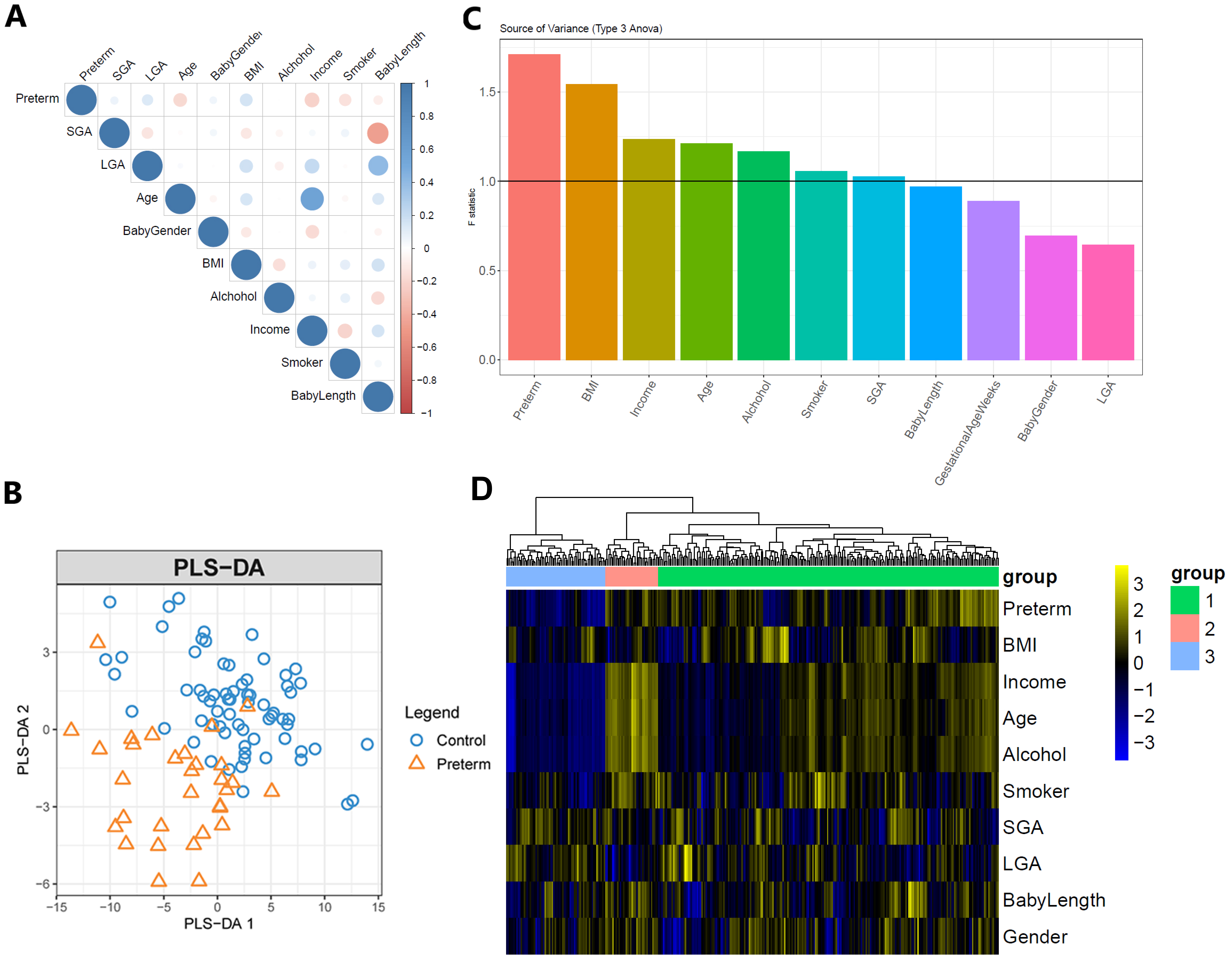

Figure 2

Click here to access/download;Figure;Fig2.pdf

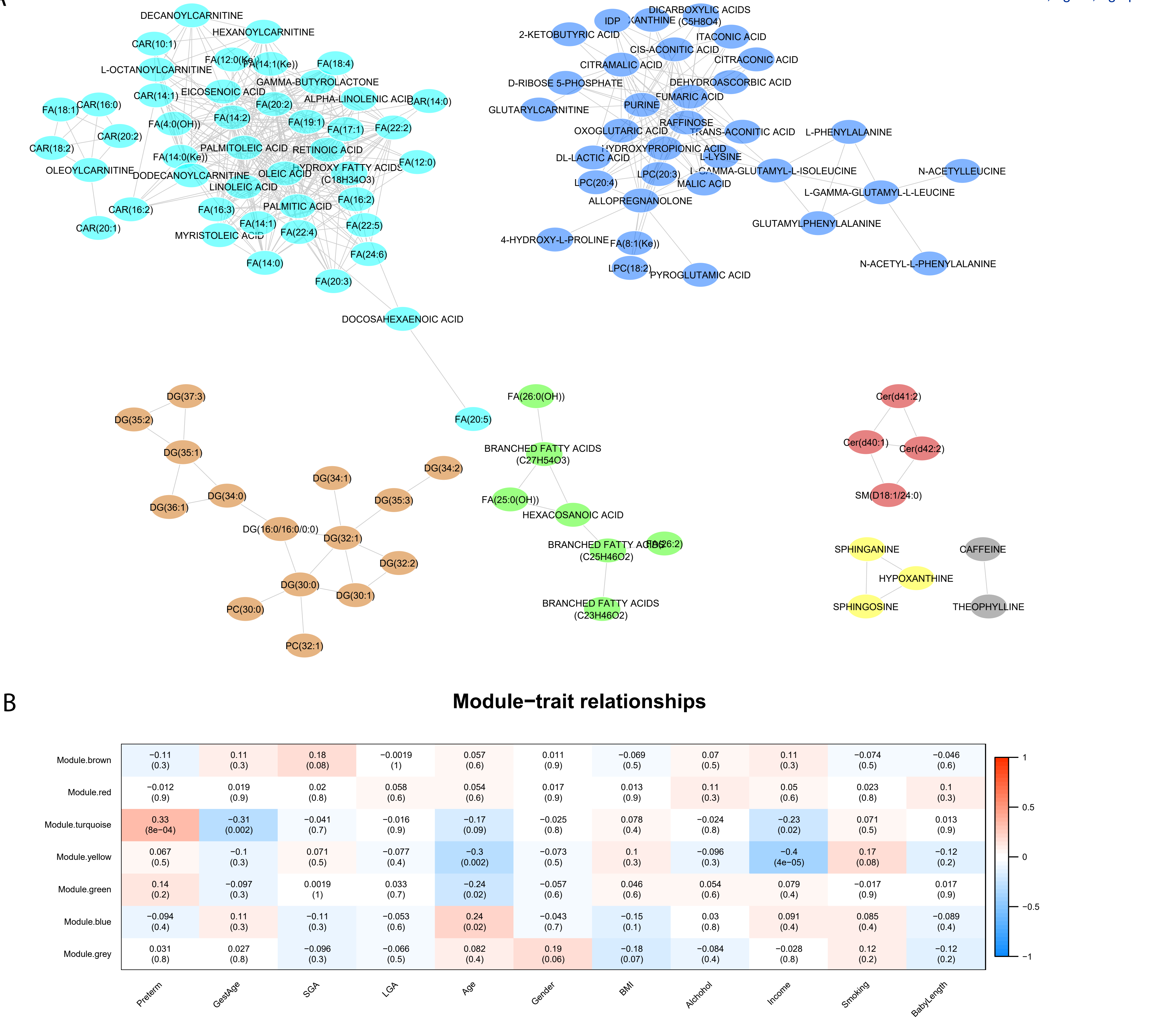

Figure3

Click here to access/download;Figure;Fig3.pdf

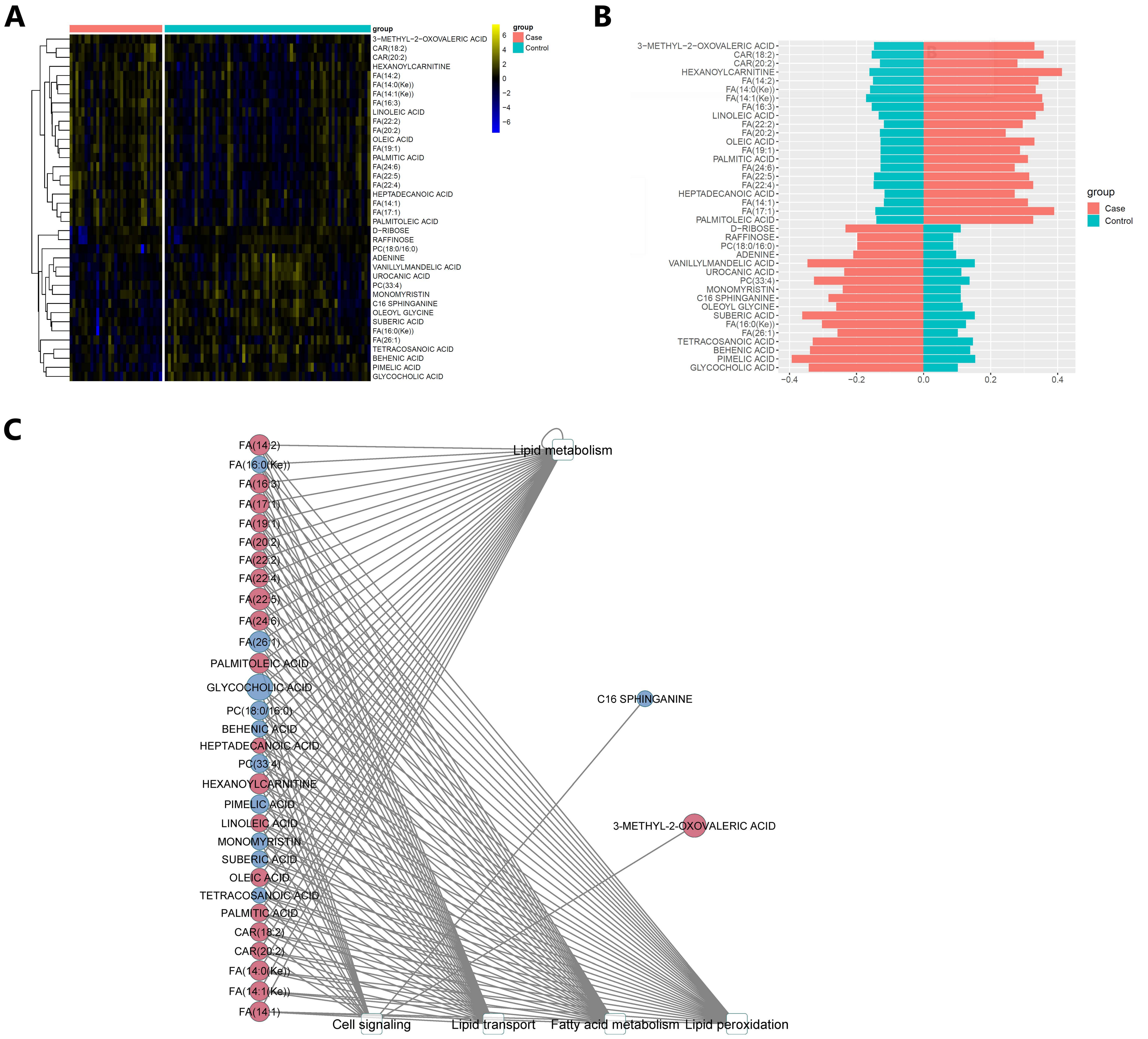

Figure4
Click here to access/download;Figure;Fig4.pdf

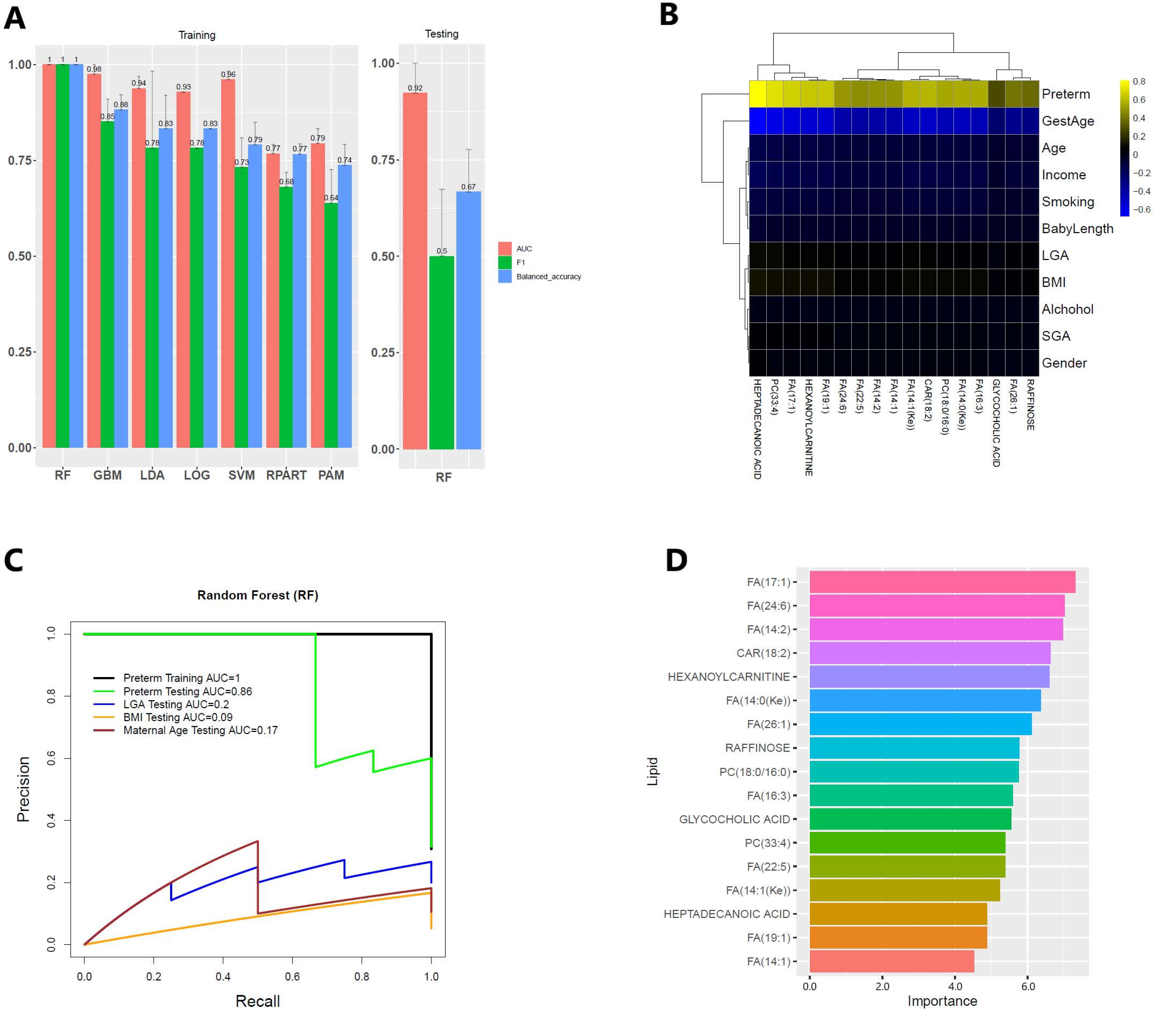

Figure5

Click here to access/download;Figure;Fig5.pdf

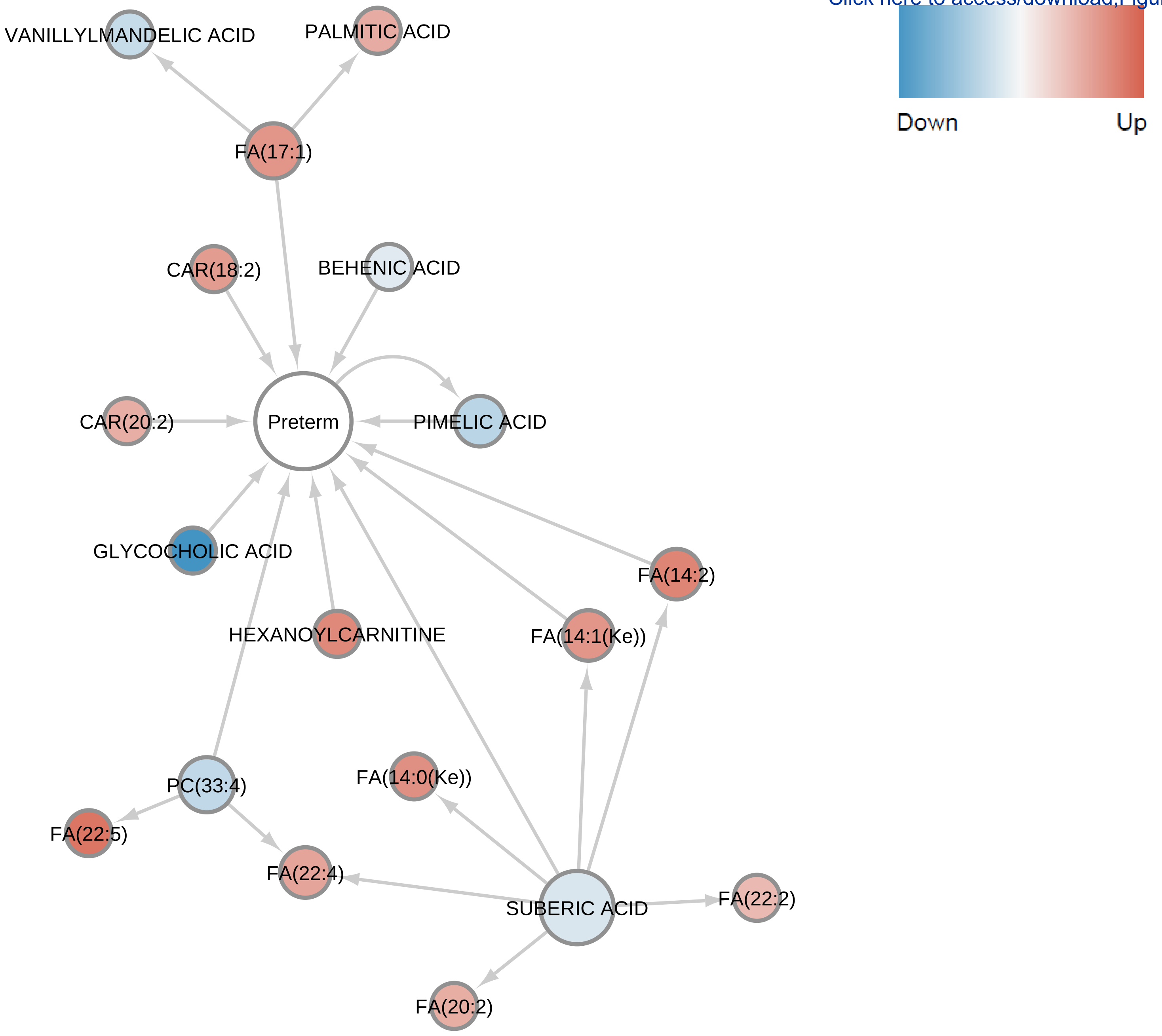

Figure6

Click here to access/download;Figure;Fig6.pdf

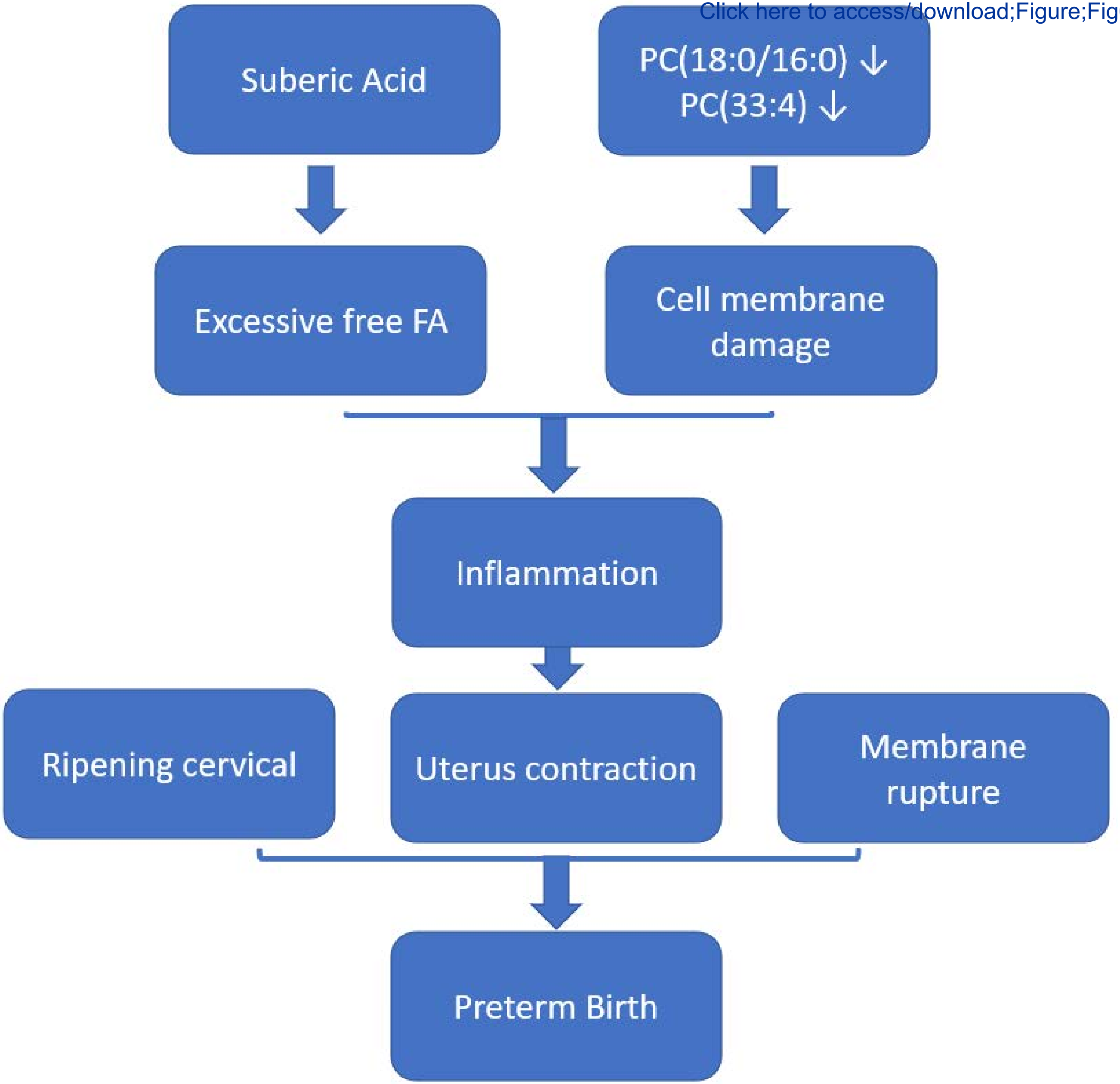

Table S1

Click here to access/download
Supplementary Material
Table S1.pdf

Supplementary FigureS1

Click here to access/download
Supplementary Material
FigS1.pdf

Supplementary FigureS2

Click here to access/download
Supplementary Material
FigS2.pdf

Supplementary FigureS3

Click here to access/download
Supplementary Material
FigS3.pdf

Supplementary FigureS4

Click here to access/download
Supplementary Material
FigS4.pdf
