# Supplementary material for: Maternal plasma lipids are involved in the pathogenesis of preterm birth
Source: Gigascience. 2022 Feb 15;11:giac004. doi: 10.1093/gigascience/giac004 (PMC8847704; doi:10.1093/gigascience/giac004)
Supplement: giac004_Supplemental_Figures_and_Table [file giac004_supplemental_figures_and_table.zip › Table S1.pdf]

Table S1. Log Fold Changes (logFC) of 38 metabolites found in the differential analysis.

	**logFC**
**HEXANOYLCARNITINE**	0.472
**3-METHYL-2-OXOVALERIC ACID**	0.680
**FA(17:1)**	0.402
**VANILLYLMANDELIC ACID**	-0.305
**PC(33:4)**	-0.332
**SUBERIC ACID**	-0.224
**FA(16:0(Ke))**	-0.224
**FA(22:5)**	0.574
**PIMELIC ACID**	-0.357
**TETRACOSANOIC ACID**	-0.152
**PALMITOLEIC ACID**	0.455
**BEHENIC ACID**	-0.191
**FA(14:0(Ke))**	0.434
**FA(14:1(Ke))**	0.407
**FA(22:4)**	0.332
**FA(16:3)**	0.423
**LINOLEIC ACID**	0.292
**CAR(18:2)**	0.375
**FA(14:2)**	0.492
**OLEIC ACID**	0.339
**GLYCOCHOLIC ACID**	-0.867
**FA(19:1)**	0.314
**PALMITIC ACID**	0.294
**FA(14:1)**	0.451
**MONOMYRISTIN**	-0.254
**OLEOYL GLYCINE**	-0.156
**UROCANIC ACID**	-0.148
**FA(24:6)**	0.419
**FA(22:2)**	0.221
**ADENINE**	-0.192
**FA(20:2)**	0.282
**PC(18:0/16:0)**	-0.357
**CAR(20:2)**	0.280
**FA(26:1)**	-0.520
**D-RIBOSE**	-0.381
**HEPTADECANOIC ACID**	0.167
**RAFFINOSE**	-0.550
**C16 SPHINGANINE**	-0.180
