# Supplementary figures and images for: Maternal plasma lipids are involved in the pathogenesis of preterm birth
Source: Gigascience. 2022 Feb 15;11:giac004. doi: 10.1093/gigascience/giac004 (PMC8847704; doi:10.1093/gigascience/giac004)

A

A1

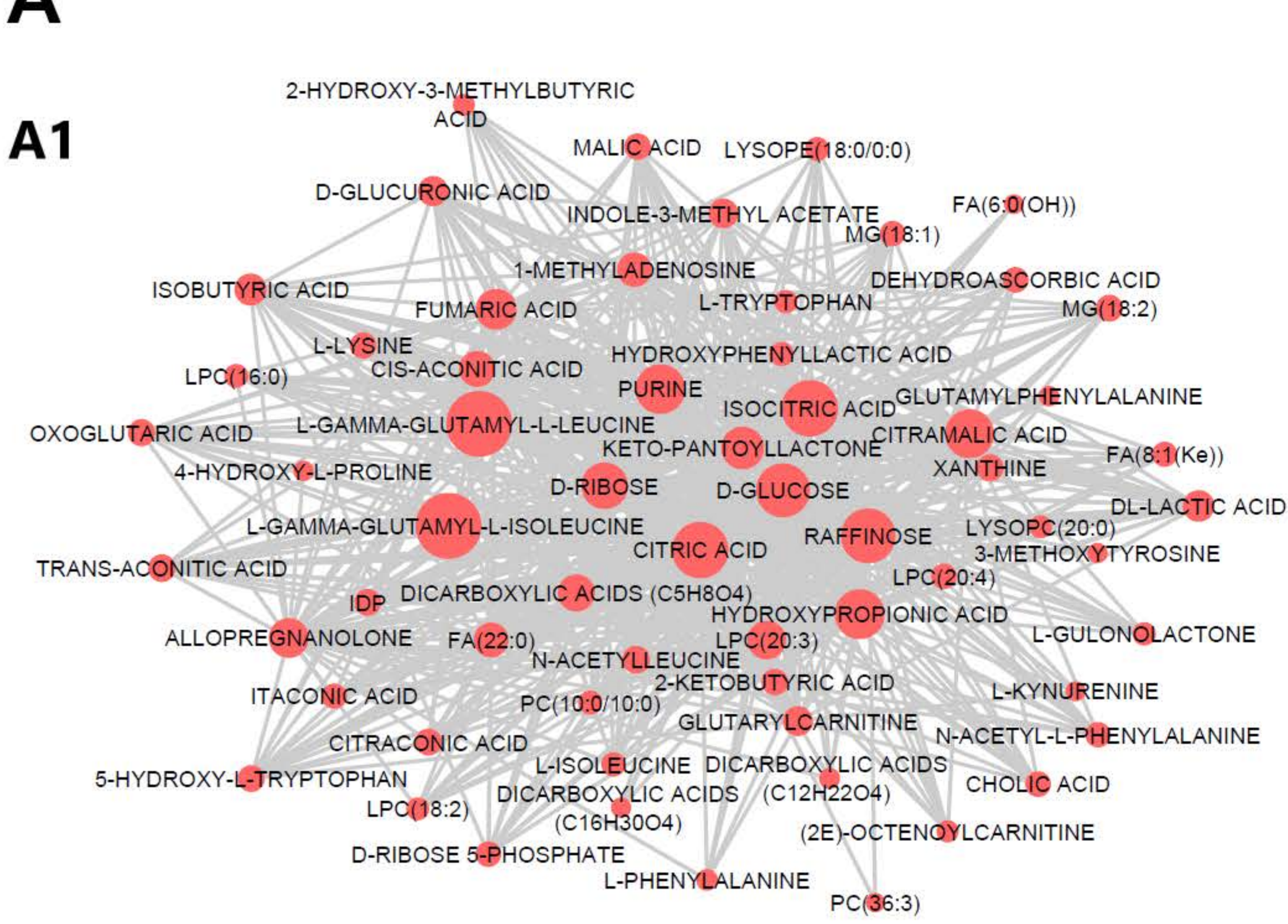

A2

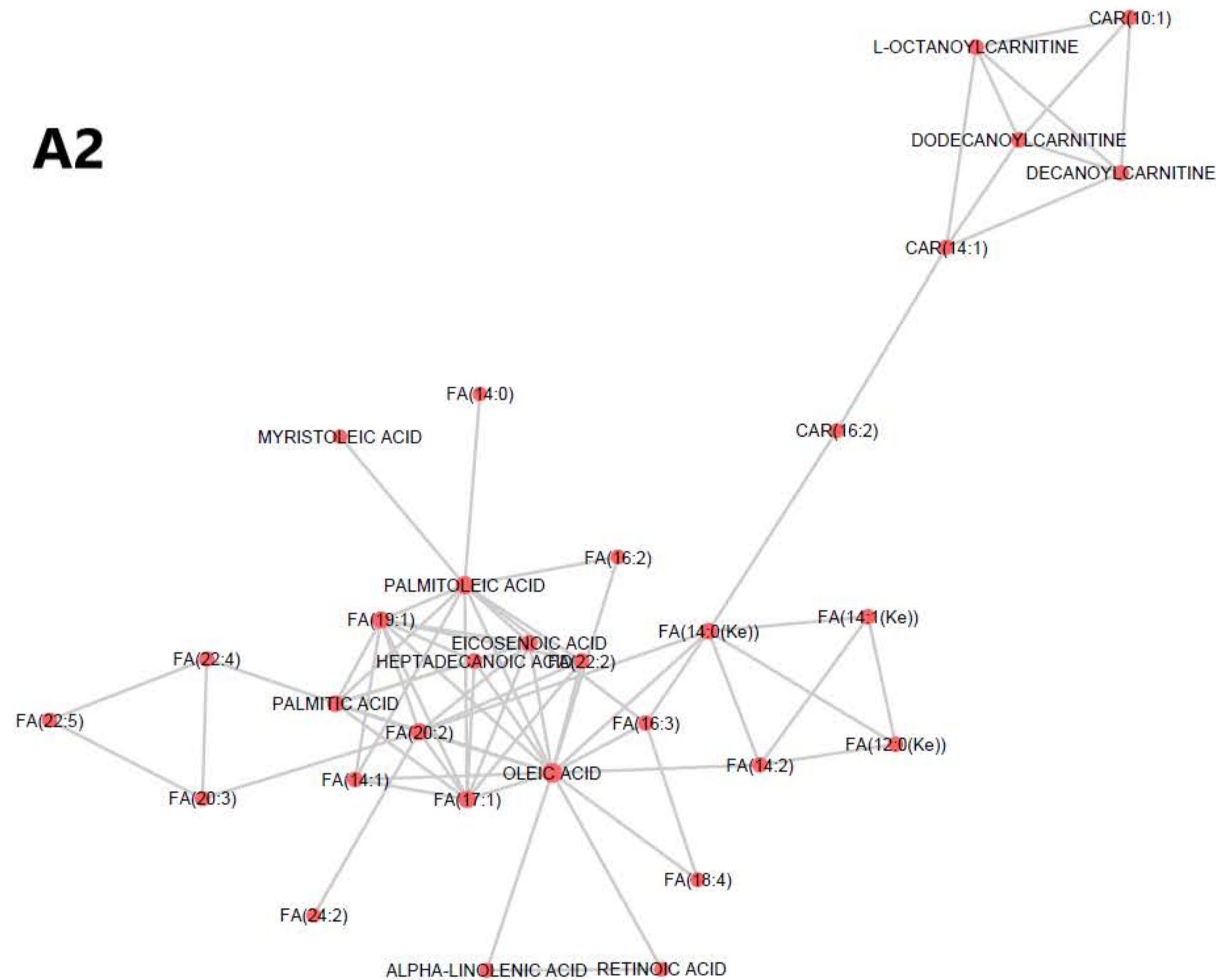

A3

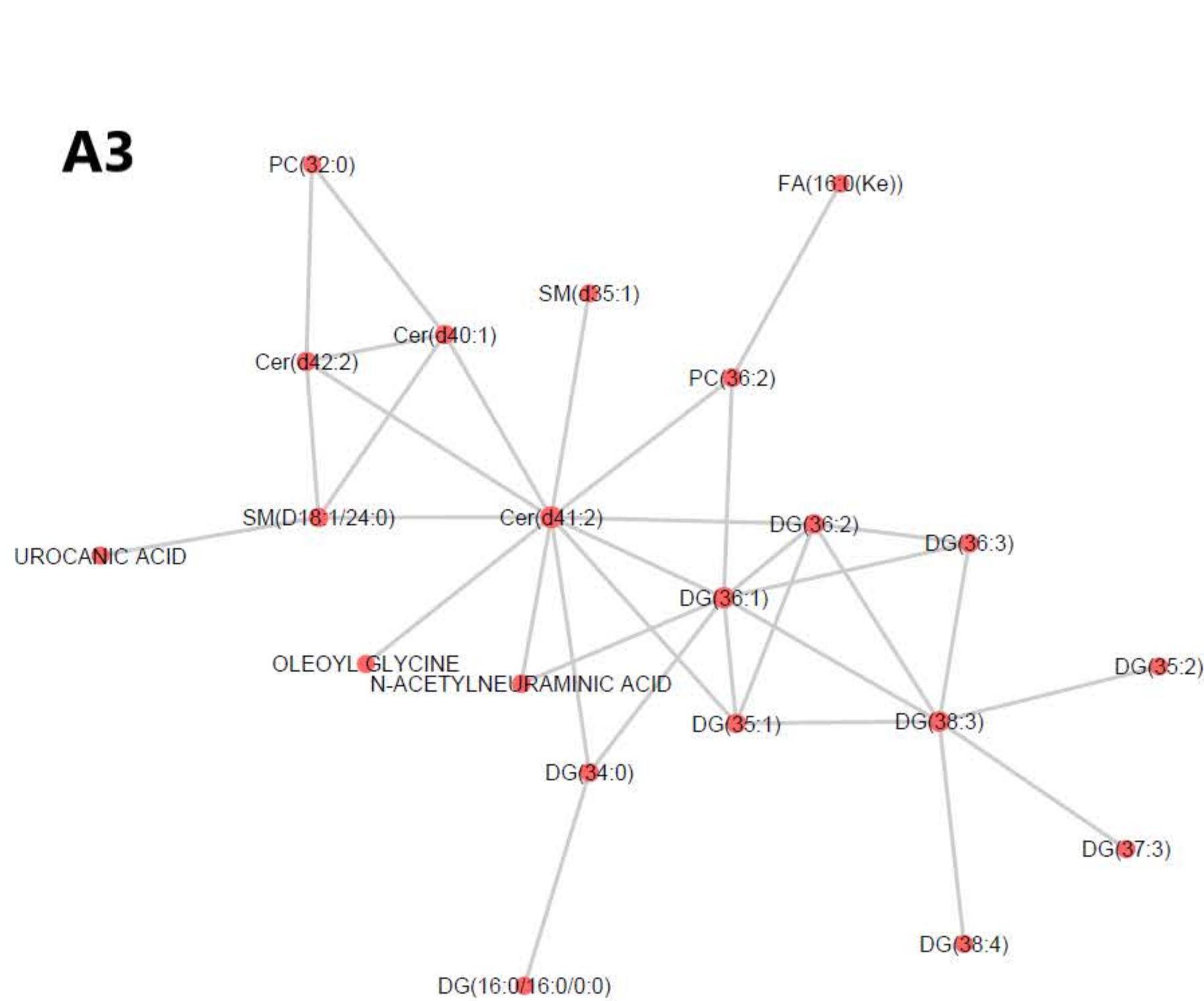

B

B1

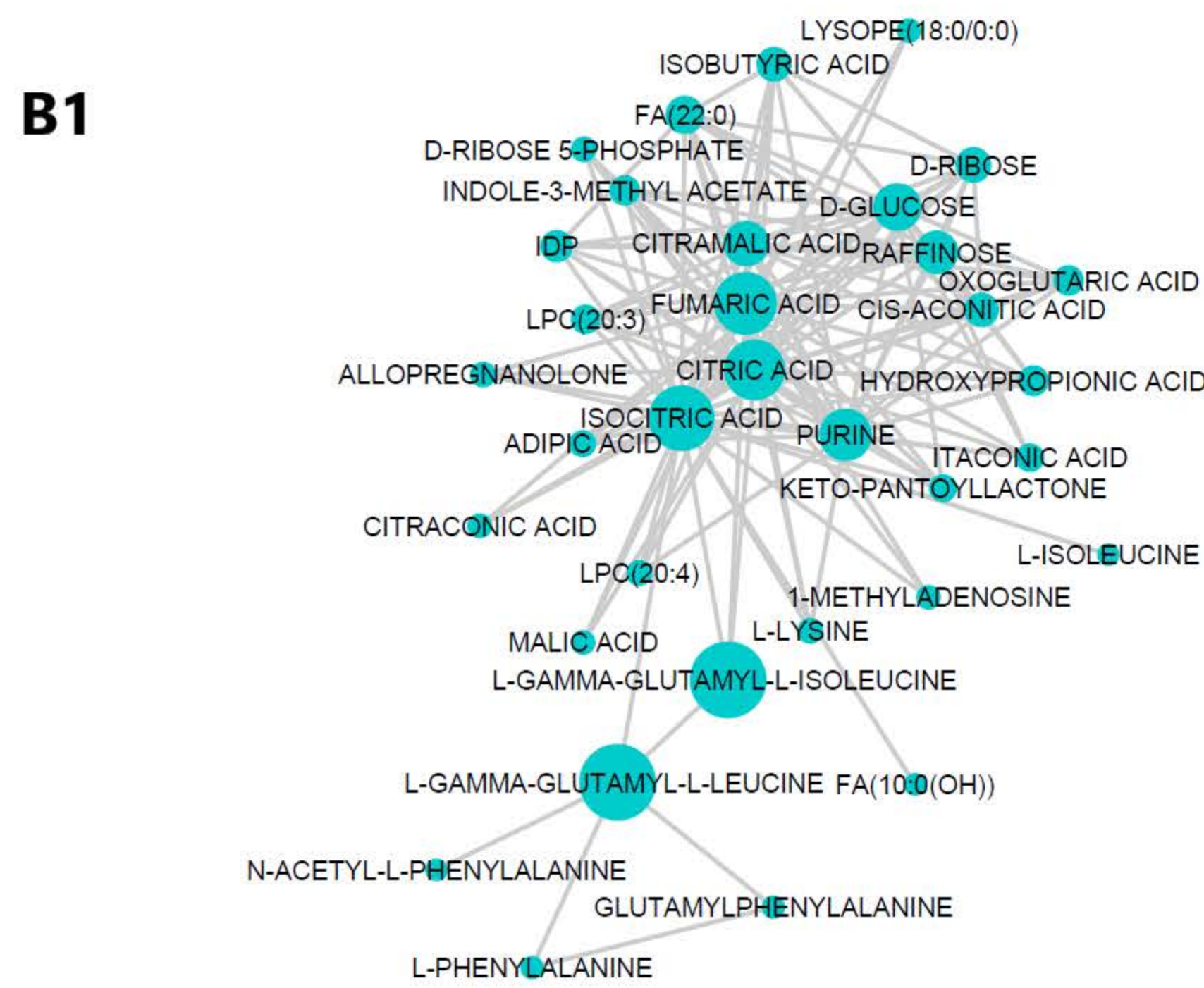

B2

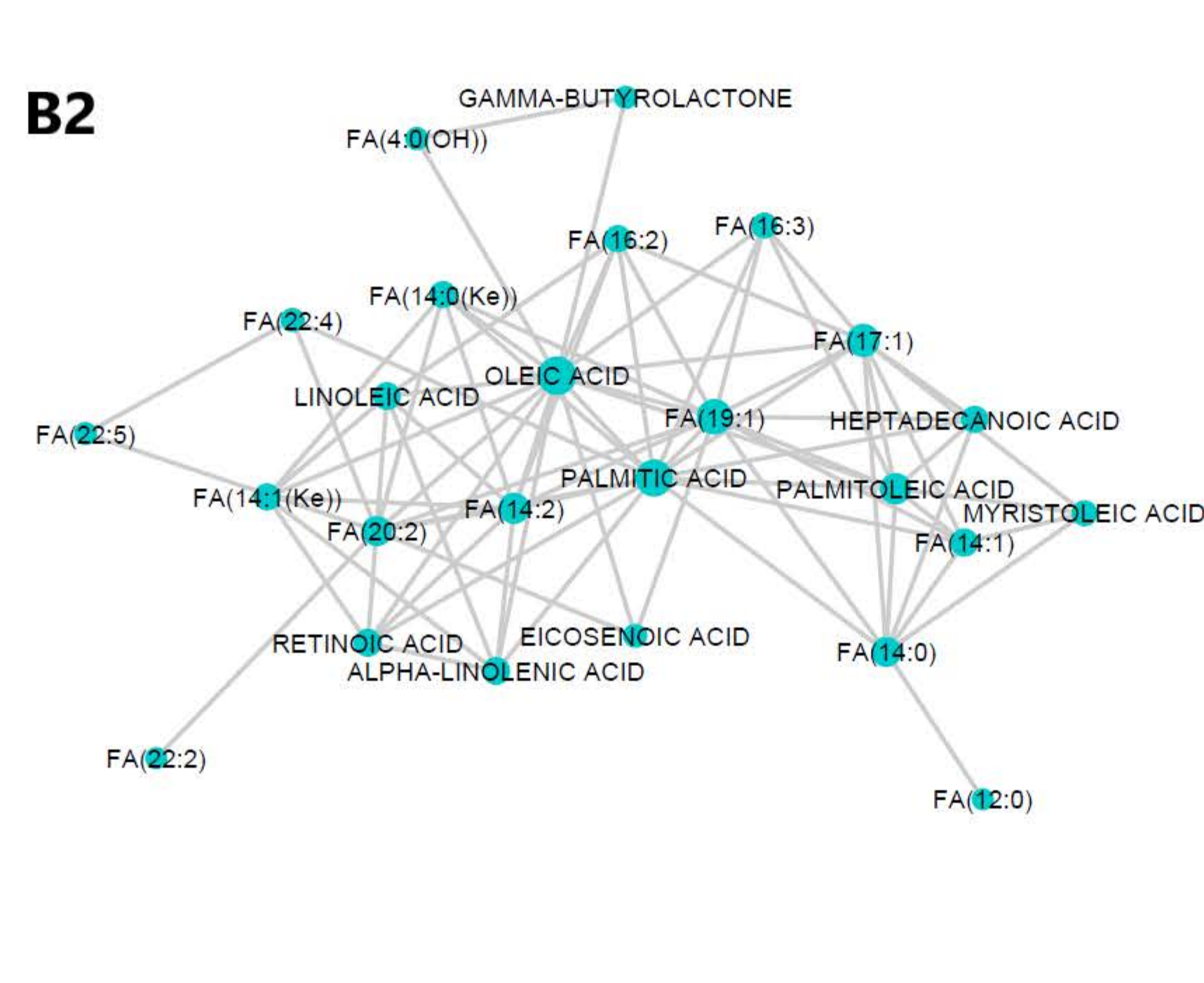

B3

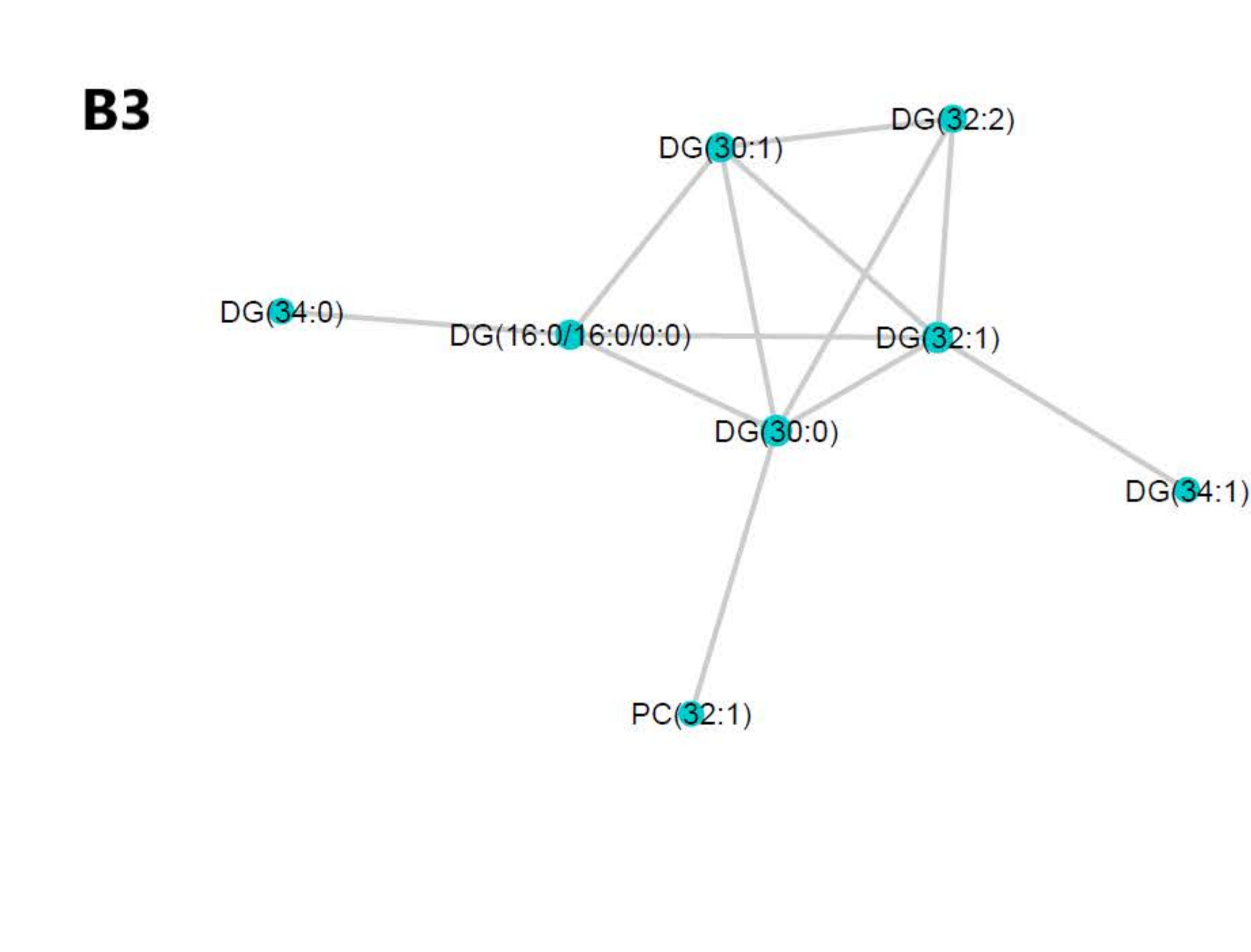

C

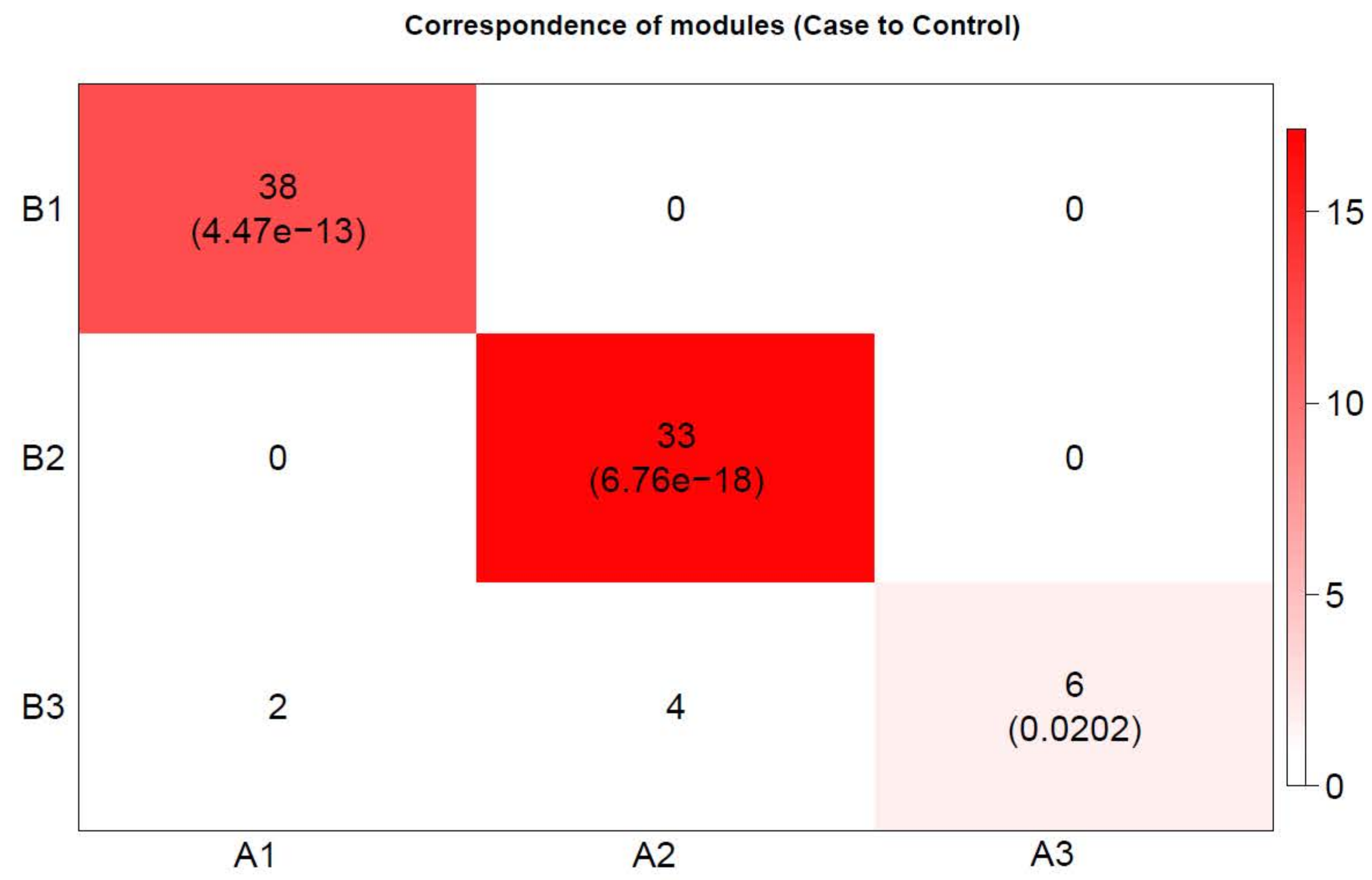

D

|                |               |         |          |
|----------------|---------------|---------|----------|
|                |               | Preterm | Controls |
| Module Density | Module 1 (38) | 0.266   | 0.169    |
|                | Module 2 (33) | 0.078   | 0.138    |

E

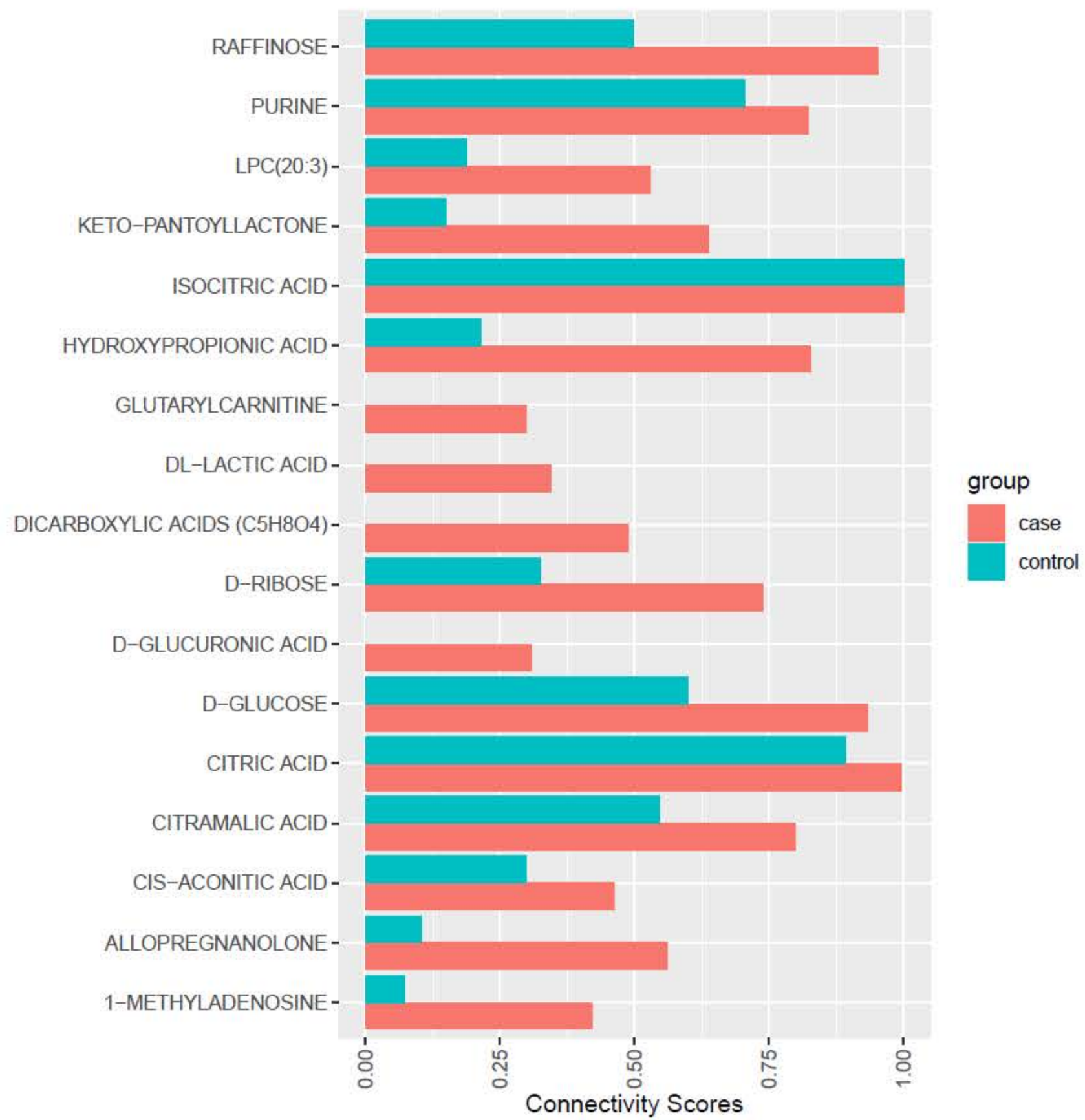

Supplement: giac004_Supplemental_Figures_and_Table [file giac004_supplemental_figures_and_table.zip › FigS1.pdf]

Fig S2 A

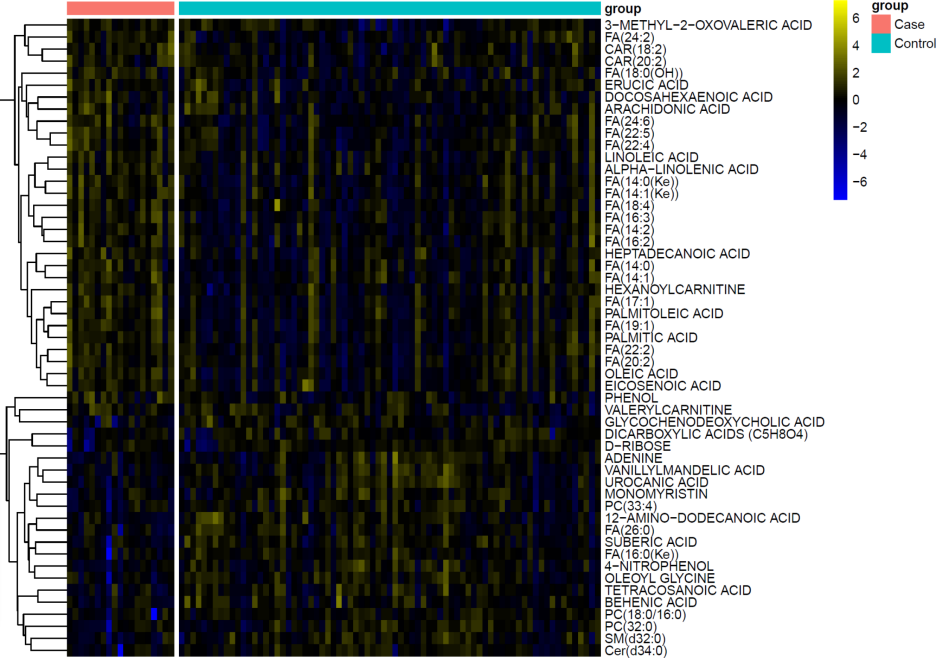

B

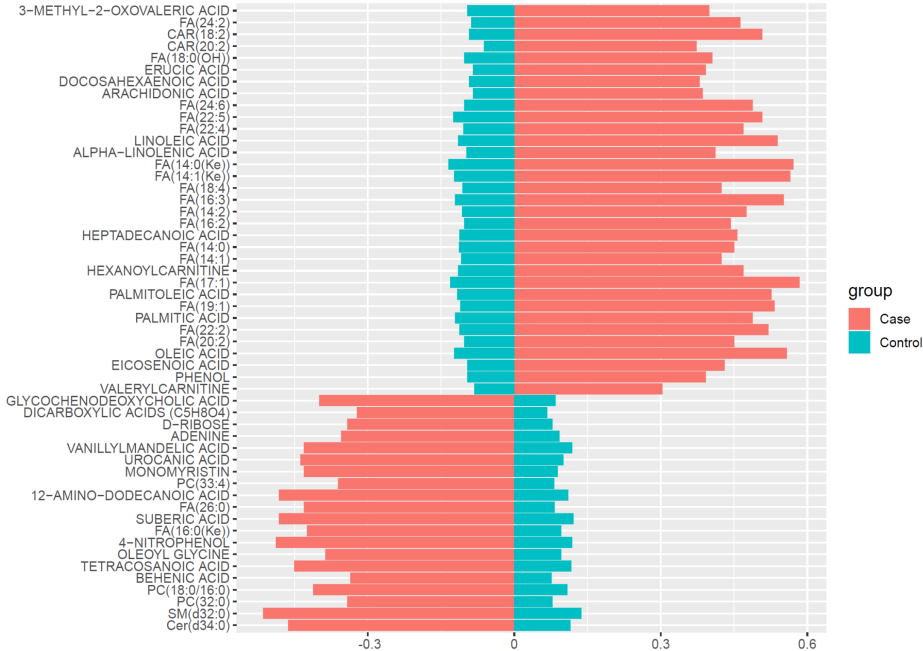

Supplement: giac004_Supplemental_Figures_and_Table [file giac004_supplemental_figures_and_table.zip › FigS2.pdf]

**Fig S3 A**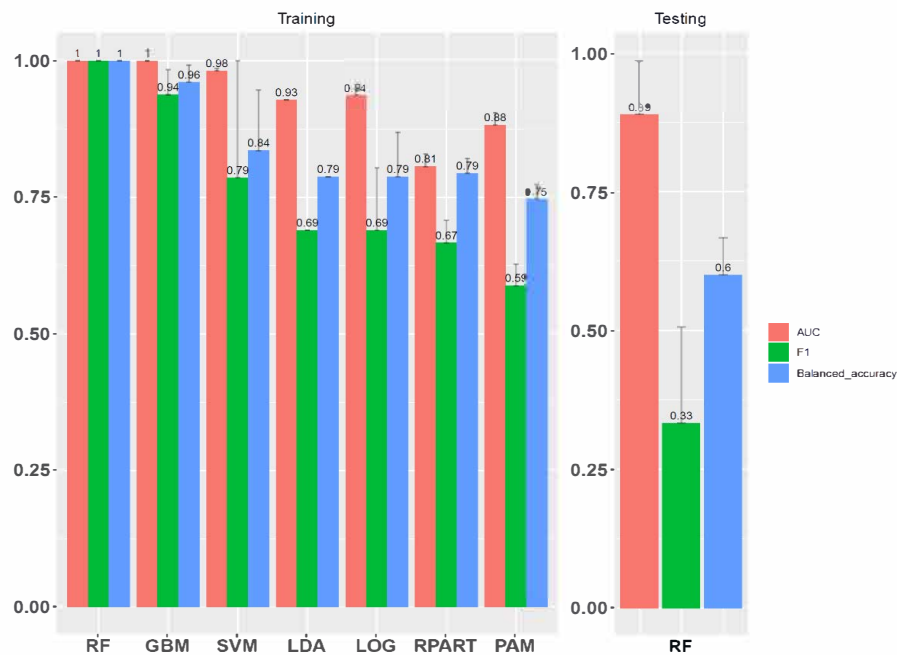**B**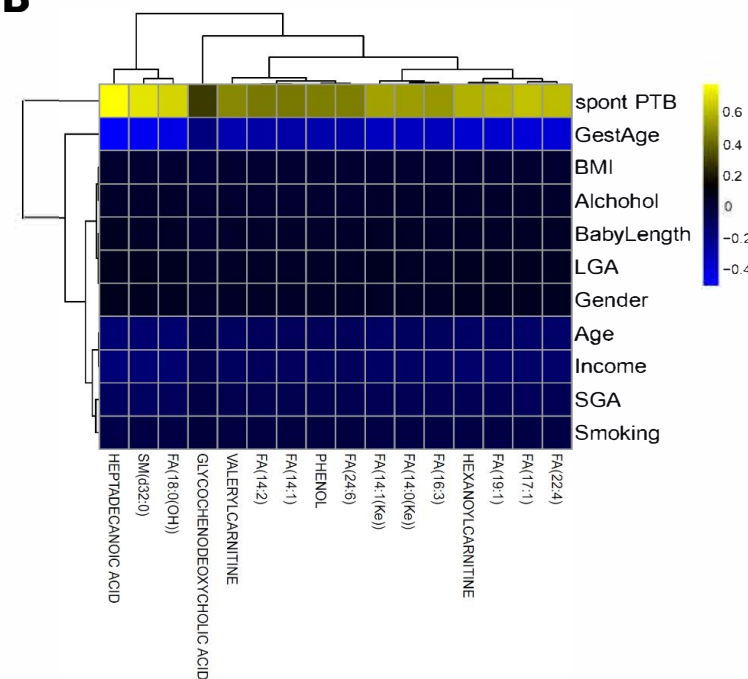**C**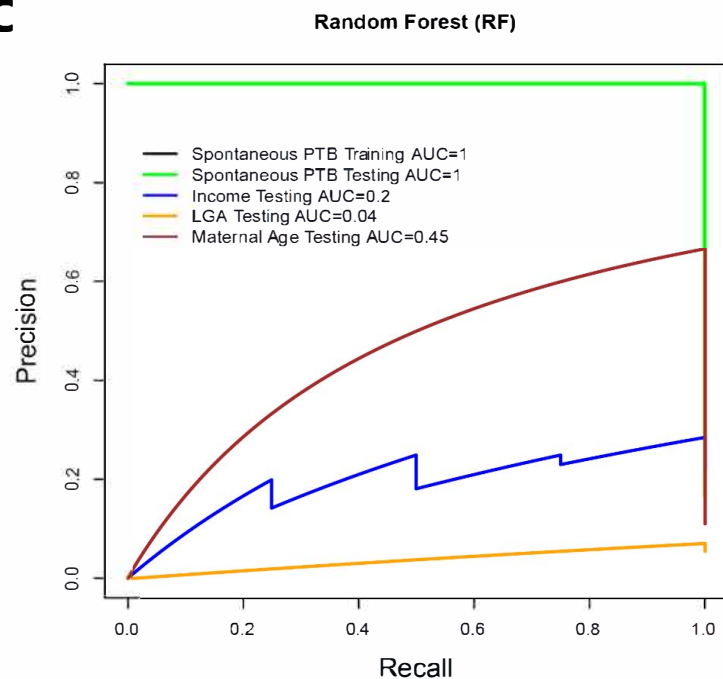**D**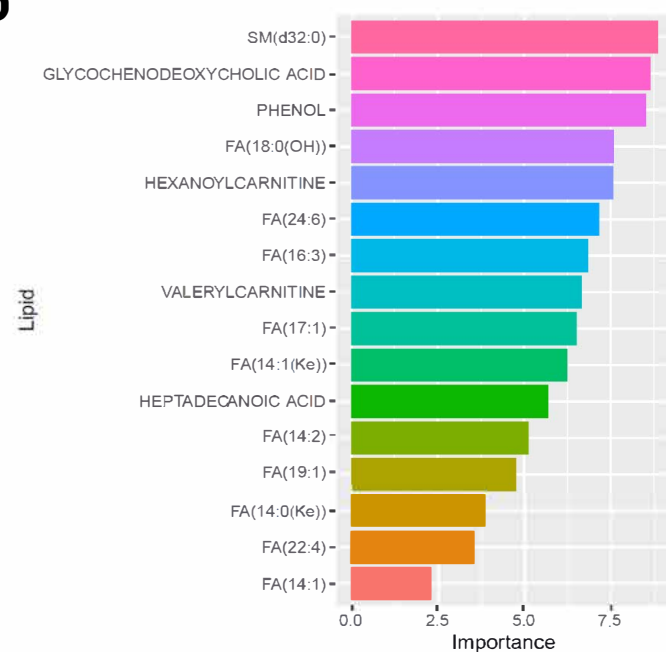

Supplement: giac004_Supplemental_Figures_and_Table [file giac004_supplemental_figures_and_table.zip › FigS3.pdf]

## Positive mode

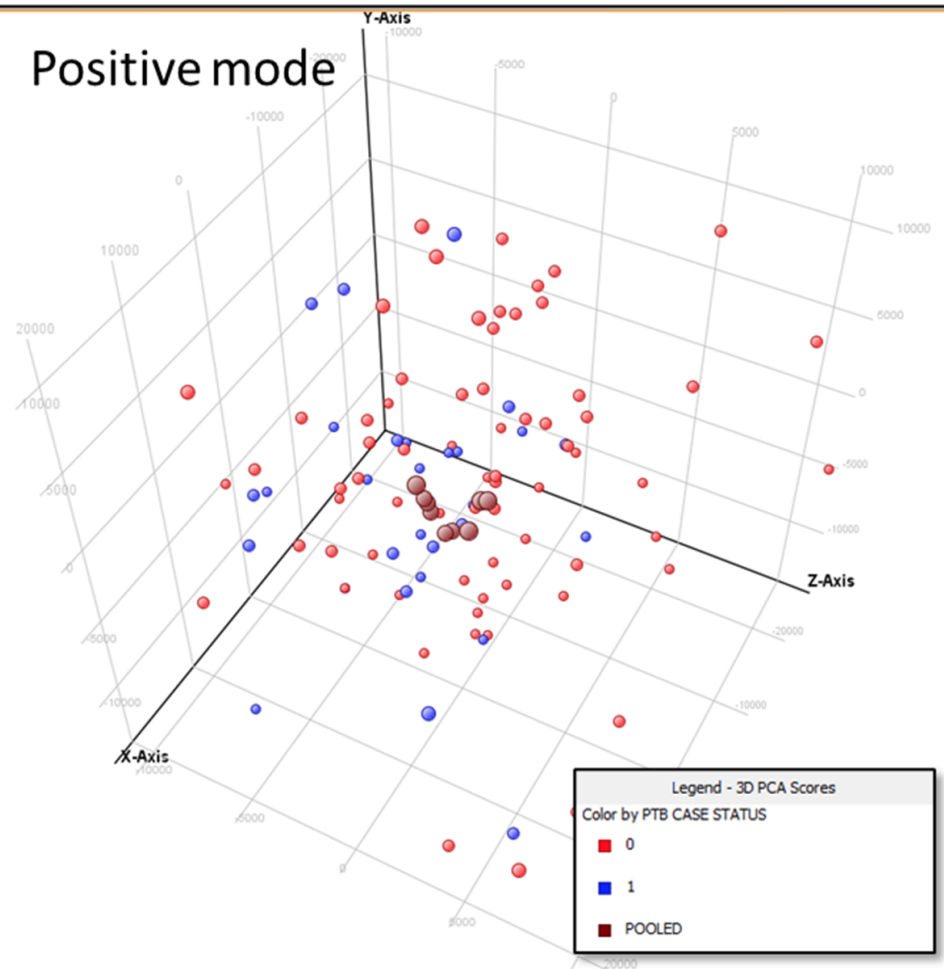

## Negative mode

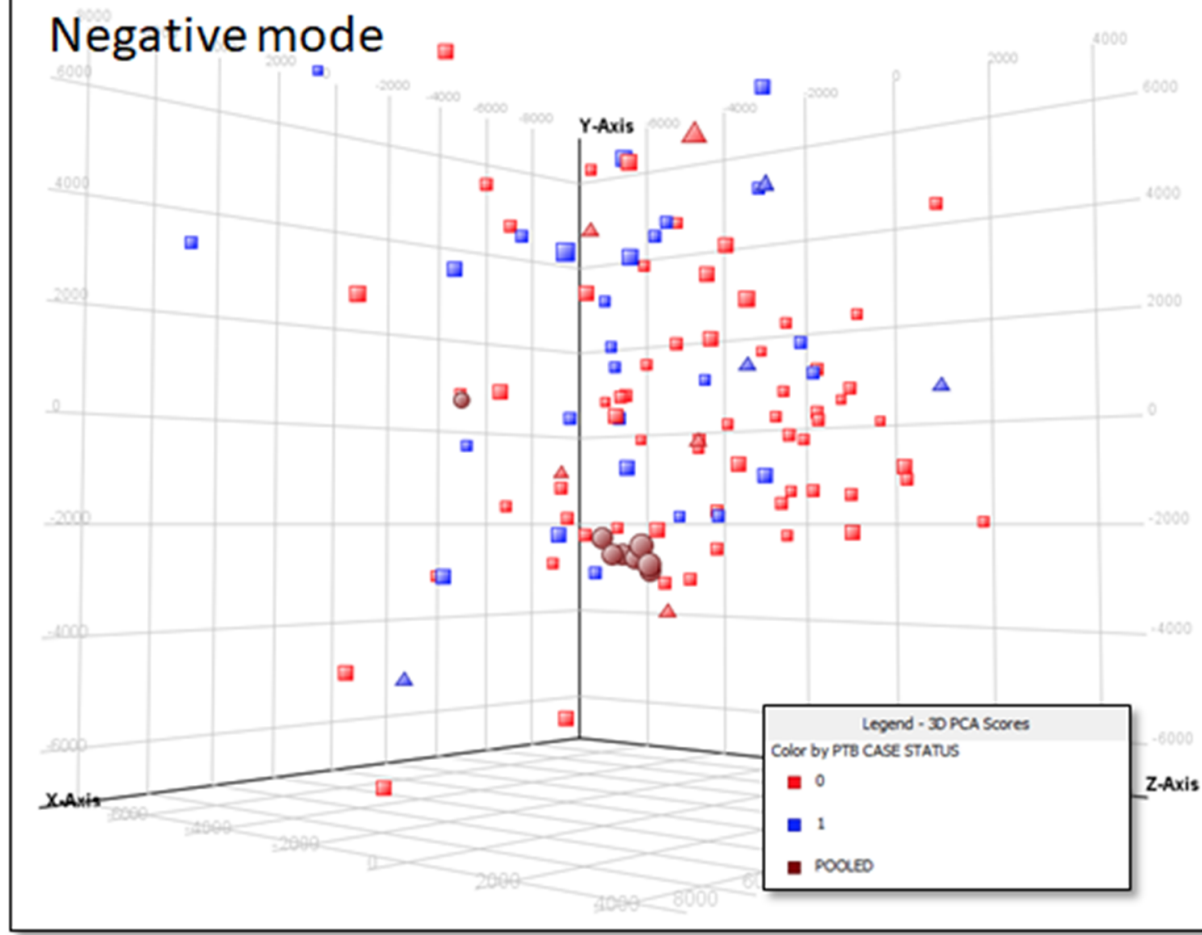

Supplement: giac004_Supplemental_Figures_and_Table [file giac004_supplemental_figures_and_table.zip › FigS4.pdf]
