# Supplementary material for: Maternal plasma lipids are involved in the pathogenesis of preterm birth
Source: Gigascience. 2022 Feb 15;11:giac004. doi: 10.1093/gigascience/giac004 (PMC8847704; doi:10.1093/gigascience/giac004)
Supplement: giac004_Response_to_Reviewer_Comments_Revision_1 [file giac004_response_to_reviewer_comments_revision_1.pdf]

Author's Response To Reviewer Comments

Close

Dear Editor,
Thanks for pointing out the potential misleading description in the method section. "The same gradient and solvent system were used for positive and negative modes of MS analysis. ". The term "same" here means the gradient. Solvent A and B are same as the previous paragraph. It's a bit misleading here so we deleted this sentence, since the following sentence describe the solvent A and B again.
Thanks
Lana

Close
