# Supplementary material for: Maternal plasma lipids are involved in the pathogenesis of preterm birth
Source: Gigascience. 2022 Feb 15;11:giac004. doi: 10.1093/gigascience/giac004 (PMC8847704; doi:10.1093/gigascience/giac004)
Supplement: giac004_Reviewer_1_Report_Original_Submission — Renato T Souza -- 7/24/2021 Reviewed [file giac004_reviewer_1_report_original_submission.pdf]

Reviewer Report

Title: Maternal plasma lipids are involved in the pathogenesis of preterm birth

Version: Original Submission **Date:** 7/24/2021

Reviewer name: Renato T Souza

Reviewer Comments to Author:

The main issue in this experiment was the use of preterm birth and not spontaneous preterm birth as the main outcome. Considering the different phenotypes related to each type of preterm birth, I would suggest analyzing those who had spontaneous PTB in separate.
- Have you considered matching case and controls according to maternal characteristics (e.g. BMI)?
- Describe in detail the study setting: single or multicenter study? The study has been conducted in Puerto Rico, right? Make it clear early in the methods.
- It is not clear whether the outcome was preterm birth or spontaneous PTB. Also, make clear what you mean by full term controls. Did you mean 39 weeks of gestation at delivery? Spontaneous delivered?
- Could you be more specific about the inclusion criteria with regard to gestational age?
- Describe the cases by BMI categories.
- What is the unit used for income?
- Some variables were described using codes that do not make sense for the audience. What do you mean by Baby gender 1? Alcohol 0, 1, 2 and 9? Please, revise. Also, give percentages.
- The proportion of smokers seems higher in the control group.
- How was small for gestational age defined?
- What procedures were taken to account for multiple comparisons testing? Have you considered calculating false discovery rates (FDR) (e.g. Benjamini-Hochberg procedure)?
- The samples were collected in a wide interval (24-28 weeks); it may generate different metabolic profiles - Have you accounted for GA at sample collection in the analysis?
- Give further detail on your mass-spectrometry analytical procedures: injector mode? Column flow mode? Stationary phase? Detector used? Mass spectra scanned?

Methods

Are the methods appropriate to the aims of the study, are they well described, and are necessary controls included? Choose an item.

Conclusions

Are the conclusions adequately supported by the data shown? Choose an item.

Reporting Standards

Does the manuscript adhere to the journal's guidelines on [minimum standards of reporting?]() Choose an item.

Choose an item.

Statistics

Are you able to assess all statistics in the manuscript, including the appropriateness of statistical tests used? Choose an item.

Quality of Written English

Please indicate the quality of language in the manuscript: Choose an item.

Declaration of Competing Interests

Please complete a declaration of competing interests, considering the following questions:

- Have you in the past five years received reimbursements, fees, funding, or salary from an organisation that may in any way gain or lose financially from the publication of this manuscript, either now or in the future?
- Do you hold any stocks or shares in an organisation that may in any way gain or lose financially from the publication of this manuscript, either now or in the future?
- Do you hold or are you currently applying for any patents relating to the content of the manuscript?
- Have you received reimbursements, fees, funding, or salary from an organization that holds or has applied for patents relating to the content of the manuscript?
- Do you have any other financial competing interests?
- Do you have any non-financial competing interests in relation to this paper?

If you can answer no to all of the above, write 'I declare that I have no competing interests' below. If your reply is yes to any, please give details below.

none

I agree to the open peer review policy of the journal. I understand that my name will be included on my report to the authors and, if the manuscript is accepted for publication, my named report including any attachments I upload will be posted on the website along with the authors' responses. I agree for my report to be made available under an Open Access Creative Commons CC-BY license (http://creativecommons.org/licenses/by/4.0/). I understand that any comments which I do not wish to be included in my named report can be included as confidential comments to the editors, which will not be published.

Choose an item.

To further support our reviewers, we have joined with Publons, where you can gain additional credit to further highlight your hard work (see: https://publons.com/journal/530/gigascience). On publication of this paper, your review will be automatically added to Publons, you can then choose whether or not to claim your Publons credit. I understand this statement.

Yes Choose an item.
