# Supplementary material for: Maternal plasma lipids are involved in the pathogenesis of preterm birth
Source: Gigascience. 2022 Feb 15;11:giac004. doi: 10.1093/gigascience/giac004 (PMC8847704; doi:10.1093/gigascience/giac004)
Supplement: giac004_Reviewer_2_Report_Original_Submission — Xiangping Lin -- 8/25/2021 Reviewed [file giac004_reviewer_2_report_original_submission.pdf]

Reviewer Report

Title: Maternal plasma lipids are involved in the pathogenesis of preterm birth

Version: Original Submission **Date:** 8/25/2021

Reviewer name: Xiangping Lin

Reviewer Comments to Author:

The authors report an untargeted LC-MS based metabolomics study, which characterizes maternal plasma of 31 preterm birth women and 69 full-term labor controls at 24-28 gestational weeks, and proposed a panel of 17 preterm biomarkers that are involved in cell signaling, lipid metabolism, and lipid peroxidation functions. The authors conclude that lipids, particularly fatty acids, are involved in the pathogenesis of preterm birth. The present study is on a topic of relevance and the concept of the study and some of the findings may be interested to the readers of the journal.

In general, I found the paper to be overall well organized, with sufficient data presented, and much of it to be well described. However, I hope the authors could share more details on the below points:

1. What is the resolution of MS technique reported in this study?
2. During the LC-MS data processing, was the blank subtraction performed? More information is needed on LC-MS data post-processing.
3. The authors descripted that the LC-MS analysis was performed both in positive and in negative ion mode, for these 333 metabolites detected, how many metabolites detected in positive mode and how many of them detected in negative ion mode? In this study, it looks that the positive LC-MS data and negative LC-MS data were combined together for further statistical analysis, if this is the case, how does this combination work?
4. Regarding metabolomics data quality check, did author performed a PCA with QC samples?
5. For the PLS-DA model (Fig. 1B), how many components does the model have? What are the R2 and Q2 value of the model?
6. Regarding the 17 preterm metabolites, are there any fold changes in these metabolites?

Methods

Are the methods appropriate to the aims of the study, are they well described, and are necessary controls included? Choose an item.

Conclusions

Are the conclusions adequately supported by the data shown? Choose an item.

Reporting Standards

Does the manuscript adhere to the journal's guidelines on [minimum standards of reporting?]() Choose an item.

Choose an item.

Statistics

Are you able to assess all statistics in the manuscript, including the appropriateness of statistical tests used? Choose an item.

Quality of Written English

Please indicate the quality of language in the manuscript: Choose an item.

Declaration of Competing Interests

Please complete a declaration of competing interests, considering the following questions:

- Have you in the past five years received reimbursements, fees, funding, or salary from an organisation that may in any way gain or lose financially from the publication of this manuscript, either now or in the future?
- Do you hold any stocks or shares in an organisation that may in any way gain or lose financially from the publication of this manuscript, either now or in the future?
- Do you hold or are you currently applying for any patents relating to the content of the manuscript?
- Have you received reimbursements, fees, funding, or salary from an organization that holds or has applied for patents relating to the content of the manuscript?
- Do you have any other financial competing interests?
- Do you have any non-financial competing interests in relation to this paper?

If you can answer no to all of the above, write 'I declare that I have no competing interests' below. If your reply is yes to any, please give details below.

I declare that I have no competing interests.

I agree to the open peer review policy of the journal. I understand that my name will be included on my report to the authors and, if the manuscript is accepted for publication, my named report including any attachments I upload will be posted on the website along with the authors' responses. I agree for my report to be made available under an Open Access Creative Commons CC-BY license (http://creativecommons.org/licenses/by/4.0/). I understand that any comments which I do not wish to be included in my named report can be included as confidential comments to the editors, which will not be published.

Choose an item.

To further support our reviewers, we have joined with Publons, where you can gain additional credit to further highlight your hard work (see: https://publons.com/journal/530/gigascience). On publication of this paper, your review will be automatically added to Publons, you can then choose whether or not to claim your Publons credit. I understand this statement.

Yes Choose an item.
